# Comprehensive analysis of expression and prognostic value of the claudin family in human breast cancer

**DOI:** 10.18632/aging.202687

**Published:** 2021-03-10

**Authors:** Guangda Yang, Liumeng Jian, Qianya Chen

**Affiliations:** 1 Department of Cancer Chemotherapy, Zengcheng District People’s Hospital of Guangzhou, Guangdong Province, China; 2 Department of Neurology, Zengcheng District People’s Hospital of Guangzhou, Guangdong Province, China

**Keywords:** claudins, breast cancer, prognosis, ONCOMINE, bc-GenExMiner v4.3

## Abstract

Claudins (CLDN) are structural components of tight junctions that function in paracellular transport and maintain the epithelial barrier function. Altered expression and distribution of members of the claudin family have been implicated in several cancers including breast cancer (BC). We performed a comprehensive analysis of the expression and prognostic value of claudins in BC using various online databases. Compared with normal tissues, CLDN3, 4, 6, 7, 9, and 14 were upregulated in BC tissues, whereas CLDN2, 5, 8, 10, 11, 15, 19, and 20 were downregulated. A high expression of CLDN2, 5, 6, 9, 10, 11, and 14–20 was associated with better relapse-free survival (RFS), whereas a high CLDN3 expression correlated with poor RFS. In addition, a high expression of CLDN3, 4, 14, and 20 was associated with poor overall survival (OS), whereas that of CLDN5 and CLDN11 was linked to a better OS. Although METABRIC and TCGA datasets revealed 22% and 27% gene alterations, respectively, in the members of the claudin family, these were not associated with survival. These findings suggest CLDN3, 5, and 11 could be promising therapeutic targets for patients with BC.

## INTRODUCTION

Breast cancer (BC) is one of the most commonly diagnosed cancers worldwide. Although its incidence rates have declined continuously due to improvements in treatment strategies and early detection, BC is still the second leading cause of cancer-related deaths among women [1]. BC is a genetically heterogeneous group of tumors with a variety of morphologic features and is classified into four distinct molecular subtypes based on the immunohistochemical expression of estrogen receptor (ER), progesterone receptor (PR), and human epidermal growth factor receptor 2 (Her2): ER/PR+, Her2– (Luminal A); ER/PR+, Her2+ (Luminal B); ER/PR–, Her2+ (HER2+); and ER/PR–, Her2– (basal-like/triple-negative breast cancer [TNBC]) [2]. Endocrine therapy can improve the survival rate of patients with luminal subtype BC, whereas trastuzumab is effective against Her2+ subtype BC [3–5]. However, chemotherapy is the only available treatment approach against basal-like BC and TNBC due to the lack of effective biomarkers. Interestingly, a novel claudin-low molecular subtype of BC has been recently identified [6], characterized by low expression of tight junction and epithelial cell–cell adhesion proteins, including claudin 3, 4, and 7, and E-cadherin [6]. In addition, claudin-low tumors preferentially display a triple-negative phenotype, with enhanced epithelial-to-mesenchymal transition (EMT) features, immune system responses, and stem cell-associated biological processes [7–10]. Moreover, patients with claudin-low BC have a poor overall survival (OS) when compared to those with luminal A subtype BC [10].

Claudins (CLDNs) are structural and functional components of tight junctions that regulate cellular adhesion and maintain cell polarity in epithelial and endothelial cell sheets [11, 12]. The human genome consists of 23 annotated CLDN genes (they lack CLDN13) [11, 12]. Claudins are abnormally expressed in several human cancers and could be used as promising targets for cancer detection, diagnosis, and treatment [13]. Literature reports only a few studies on the expression and functions of some claudin genes in BC ([14–33] Supplementary Table 1). We believe that understanding the expression patterns, functional roles, and prognostic values of claudins would assist in identifying potential therapeutic targets and survival biomarkers for BC.

The advent of microarray technology has revolutionized the way DNA and RNA research is conducted [34]. We comprehensively analyzed different claudin genes using various online databases to determine their expression patterns, potential functions, and distinct prognostic values in patients with BC.

## RESULTS

### Transcriptional levels of claudins in patients with breast cancer

**Figure 1:**
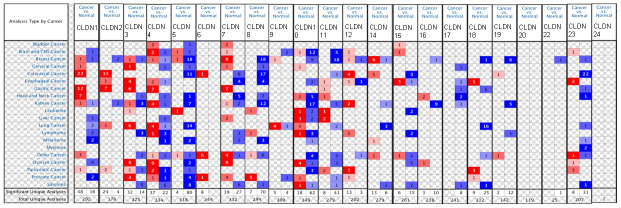
**The mRNA expression of the claudin family in different types of cancers (ONCOMINE).** Notes: The figure is generated from ONCOMINE with exact thresholds (*p*-value: 0.05; fold change: 2; gene rank: top 10%). The cell number represents the dataset number that meets all the thresholds, with blue for underexpression and red for overexpression. The cell color is determined by the best gene rank percentile for the analyses within the cell.

We first compared the mRNA levels of claudins in BC and the corresponding normal samples using the ONCOMINE database (Figure 1 and Supplementary Table 2). Details of major datasets of the claudin family in BC are shown in Supplementary Table 2 [35–46]. In total, we identified 22 claudins in BC samples.

The results indicated that the mRNA expression of CLDN2, 5, 8, 10, 11, 16, 18, 19, 22, and 23 was downregulated in BC samples (Figure 1 and Supplementary Table 2). The decreased mRNA expression of CLDN2 was found in two datasets with a fold change of –4.357 and –2.283. The mRNA expression of CLDN5 was only upregulated in one study but downregulated in 18 studies. The mRNA expression of CLDN8 was downregulated with a fold change ranging from –2.382 to –24.488 in all 18 studies of six published datasets and the TCGA dataset. The mRNA expression of CLDN10 was downregulated with a fold change ranging from –2.117 to –7.292 in all nine studies of two published datasets and the TCGA dataset. In addition, the mRNA expression of CLDN11 was downregulated with a fold change ranging from –2.382 to –13.276 in all 18 studies of five published datasets and the TCGA dataset. The mRNA expression of CLDN19 was downregulated with a fold change ranging from –3.652 to –10.49 in all six studies of the TCGA dataset. In addition, the TCGA dataset revealed that the mRNA expression of CLDN16 (*p* = 0.001, fold change = −2.053) decreased in intraductal cribriform breast adenocarcinoma. In the BC dataset of Turashvili’s study [38], CLDN18 was downregulated in invasive ductal breast carcinoma with a fold change of –2.301 (*p* = 1.89E-04) and CLDN23 was downregulated in invasive lobular breast carcinoma with a fold change of –3.776 (*p* = 0.025). Moreover, the TCGA dataset revealed reduced mRNA expression of CLDN22 (*p* = 5.33E-06, fold change =−2.229) in invasive ductal and lobular carcinoma.

The results showed that the mRNA expression of CLDN7, 9, and 14 was upregulated in BC samples (Figure 1 and Supplementary Table 2). The mRNA expression of CLDN7 was upregulated with a fold change ranging from 2.007 to 3.625 in all eight studies of three datasets. CLDN9 was overexpressed in the BC dataset of Finak’s study with a fold change of 3.269 [40]. In addition, the mRNA expression of CLDN14 was only downregulated in one study but upregulated in six studies. However, the mRNA expression of CLDN1, 3, 4, and 12 was both overexpressed and underexpressed in one study. Unfortunately, we did not find any study on the mRNA expression of CLDN6, 15, 17, 20, and 24 in samples obtained from patients with BC and normal individuals (Figure 1 and Supplementary Table 2).

**Figure 2:**
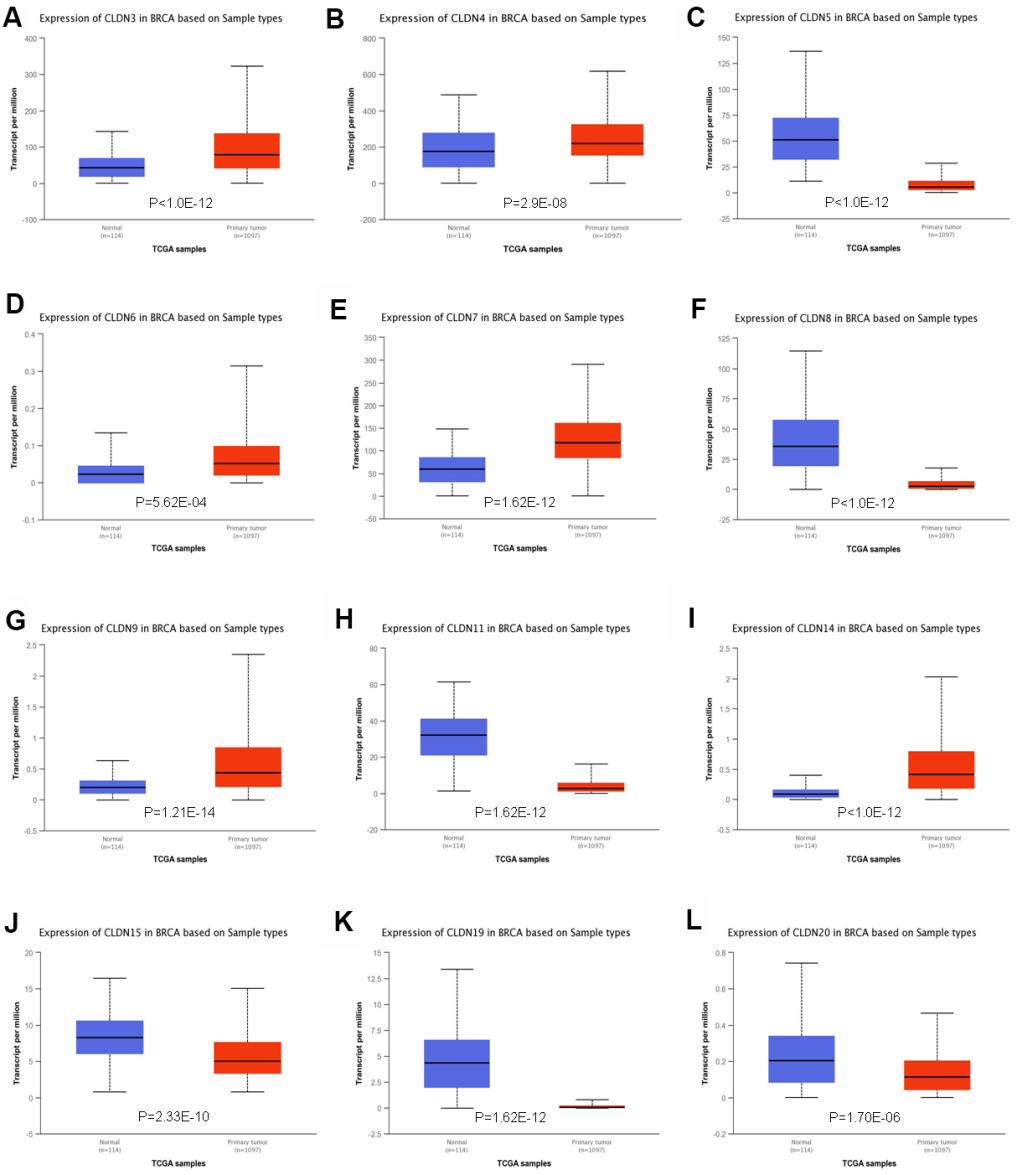
**Significant changes in claudin mRNA expression between breast cancer and normal tissues (UALCAN database).** (**A**) CLDN3; (**B**) CLDN4; (**C**) CLDN5; (**D**) CLDN6; (**E**) CLDN7; (**F**) CLDN8; (**G**) CLDN9; (**H**) CLDN11; (**I**) CLDN14; (**J**) CLDN15; (**K**) CLDN19; (**L**) CLDN20.

To further confirm the expression of members of the claudin family, we used the ULACAN database to compare the mRNA levels of claudins in the samples obtained from patients with BC with those obtained from normal individuals (Supplementary Table 3). We found that the mRNA expression of CLDN3, 4, 6, 7, 9, and 14 was higher in BC tissues than in normal tissues (Figure 2A, 2B, 2D, 2E, 2G, 2I), whereas the mRNA expression of CLDN5, 8, 11, 15, 19, and 20 was higher in normal tissues than in BC tissues (Figure 2C, 2F, 2H, 2J–2L). Therefore, comprehensive results indicated that the mRNA expression of CLDN3, 4, 6, 7, 9, and 14 was upregulated in patients with BC compared with normal individuals, whereas that of CLDN2, 5, 8, 10, 11, 15, 19, and 20 was downregulated in patients with BC. The expression of other claudin genes remains controversial.

In addition, we analyzed the promoter methylation levels of claudins in BC and normal tissues. The beta values of CLDN2, 4, 5, 6, 9, 15, 16, 17, 18, 19, and 20 indicated hypermethylation, whereas those of CLDN1, 7, 8, 10, 11, 12, and 23 indicated hypomethylation (Supplementary Table 3). However, we found that CLDN1, 5, 6, 9, 10, 11, 15, 19, and 23 had higher promoter methylation levels in BC tissues than in normal tissues, whereas CLDN2, 4, 7, 8, 12, 16, 17, 18, and 20 had lower promoter methylation levels in BC tissues than in normal tissues (Supplementary Figure 1).

### Relationship between mRNA levels of claudins and the clinicopathologic parameters of patients with breast cancer

We next analyzed the relationship between claudins and the clinicopathologic parameters of BC using the bc-GenExMiner v4.3 database (Supplementary Table 4). With respect to age, the mRNA expression of CLDN2, 8, 10, 11, 19, and 23 was low in the age group above 51 years, whereas only CLDN3 mRNA was upregulated in the older group. The mRNA expression of CLDN3, 4, 7, and 15 was upregulated, whereas that of CLDN2, 10, 19, and 23 was downregulated in the node-positive BC group. In addition, the mRNA expression of CLDN3, 5, 7, 11, and 12 was upregulated, whereas that of CLDN1, 4, 6, 8, 9, 10, 14, 16, 17, 23, and 24 was downregulated in patients positive for estrogen receptor (ER). Patients positive for progesterone receptor (PR) had a higher mRNA expression of CLDN5, 7, 11, and 12 and lower expression of CLDN1, 2, 4, 6, 8, 9, 10, 14, 16, 22, 23, and 24 mRNAs as compared with normal samples. Compared with the HER2-negative group, CLDN2 and CLDN4 mRNAs were overexpressed in the human epidermal growth factor receptor 2 (HER2)-positive group. Moreover, patients with HER2-positive BC had reduced mRNA expression of CLDN5 and CLDN12.

TNBC is a special type of BC with negative ER, PR, and HER2. Although the triple-negative status positively correlated with CLDN1, 6, 8, 9, 10, 16, 20, and 23, it had a negative correlation with CLDN3, 5, 7, 11, 12, and 19 mRNA expression. Moreover, basal-like status was positively correlated with CLDN1, 2, 4, 6, 8, 9, 10, 14, 16, 22, and 23 but negatively correlated with CLDN3, 5, 7, 11, 12, and 19 mRNA expression (Supplementary Table 4).

**Figure 3:**
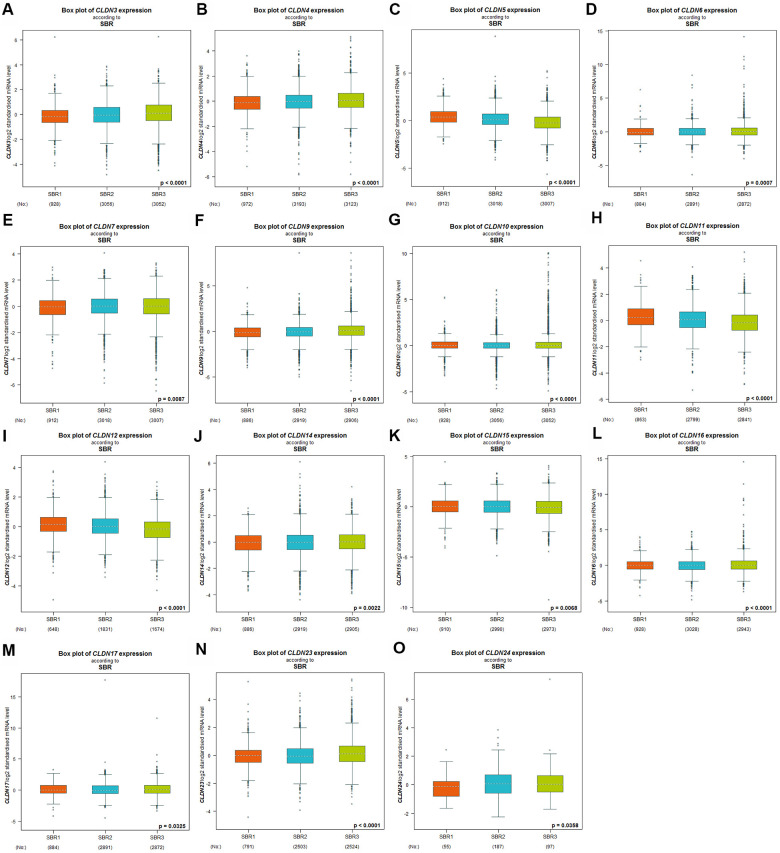
**Relationship between the claudin family and the SBR criterion.** (**A**) CLDN3; (**B**) CLDN4; (**C**) CLDN5; (**D**) CLDN6; (**E**) CLDN7; (**F**) CLDN9; (**G**) CLDN10; (**H**) CLDN11; (**I**) CLDN12; (**J**) CLDN14; (**K**) CLDN15; (**L**) CLDN16; (**M**) CLDN17; (**N**) CLDN22; (**O**) CLDN24. Global differences between the groups were assessed by Welch’s test, and *p* < 0.05 was considered significant, with Dunnett’s modified Tukey–Kramer test computed for each pairwise comparison. Abbreviations: AQP, aquaporin; NPI, Nottingham Prognostic Index; SBR, Scarff–Bloom–Richardson.

Scarff–Bloom–Richardson (SBR) grading system is considered a prognostic factor in BC. A higher SBR grade status correlated with a higher mRNA expression of CLDN3 and CLDN4 (Figure 3A, 3B) and with lower mRNA expression of CLDN5, 11, and 12 (Figure 3C, 3H, 3I). For CLDN6, 7, 9, 10, 14, 15, 16, 17, 23, and 24 (Figure 3D–3G, 3J–3O), although a substantial difference was detected in Welch’s test, certain comparison groups by Dunnett’s Tukey–Kramer test did not show a difference (the cutoff value of *p* was 0.05) (Supplementary Table 5). Other claudin genes showed no difference in the SBR grade status (Supplementary Table 5).

**Figure 4:**
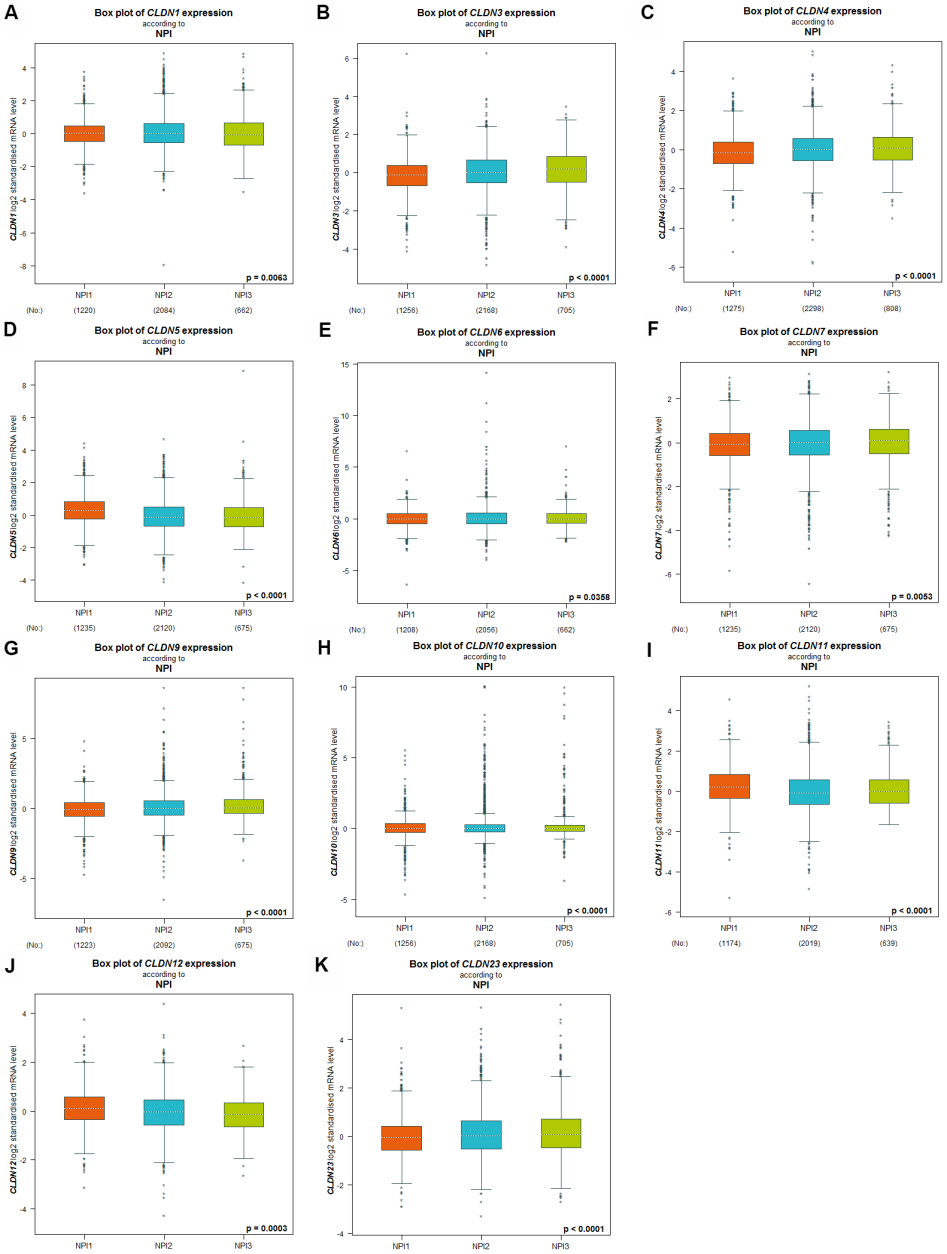
**Relationship between the Claudin family and the NPI criterion.** (**A**) CLDN1; (**B**) CLDN3; (**C**) CLDN4; (**D**) CLDN5; (**E**) CLDN6; (**F**) CLDN7; (**G**) CLDN9; (**H**) CLDN10; (**I**) CLDN11; (**J**) CLDN12; (**K**) CLDN23. Global differences between the groups were assessed by Welch’s test, and *p* < 0.05 was considered significant, with Dunnett’s Tukey–Kramer test computed for each pairwise comparison. Abbreviation: NPI, Nottingham Prognostic Index.

The Nottingham prognostic index (NPI) is another prognostic model for patients with BC. A higher NPI grade status was found to be correlated with higher mRNA expression of CLDN3 and CLDN9 (Figure 4B, 4G). For CLDN1, 4, 5, 6, 7, 10, 11, 12, and 23 (Figure 4A, 4C–4F, 4H–4K), not all pairwise comparisons in the NPI criteria were significant (*p* < 0.05) (Supplementary Table 5). Other claudin genes showed no difference in the NPI grade status (Supplementary Table 5).

### Prognostic values of claudin mRNA expression in all breast cancer groups

**Figure 5:**
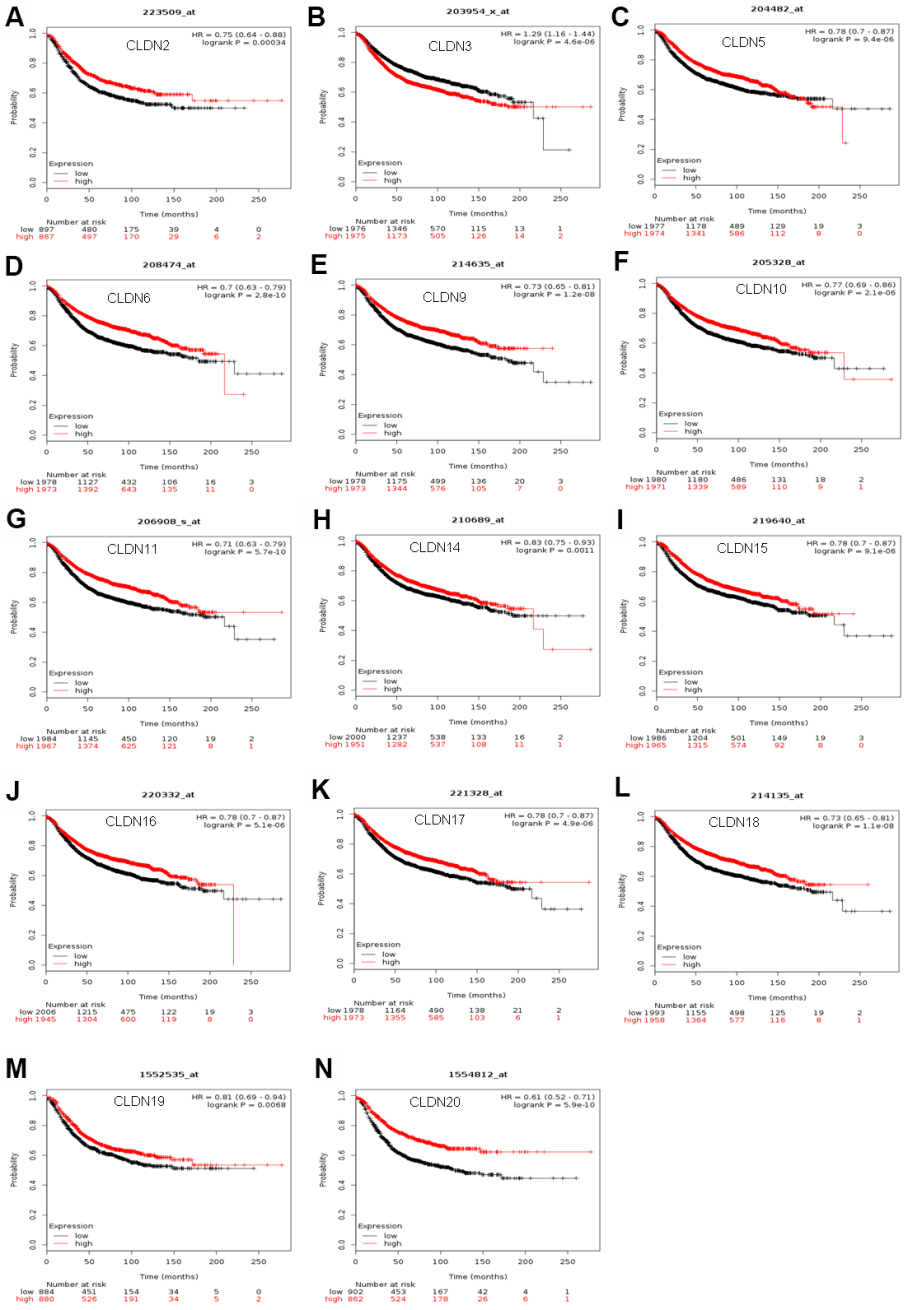
**The RFS of mRNA levels of claudins in all patients with breast cancer (Kaplan–Meier plotter).** (**A**) CLDN2; (**B**) CLDN3; (**C**) CLDN5; (**D**) CLDN6; (**E**) CLDN9; (**F**) CLDN10; (**G**) CLDN11; (**H**) CLDN14; (**I**) CLDN15; (**J**) CLDN16; (**K**) CLDN17; (**L**) CLDN18; (**M**) CLDN19; (**N**) CLDN20. Abbreviation: RFS, relapse-free survival.

**Figure 6:**
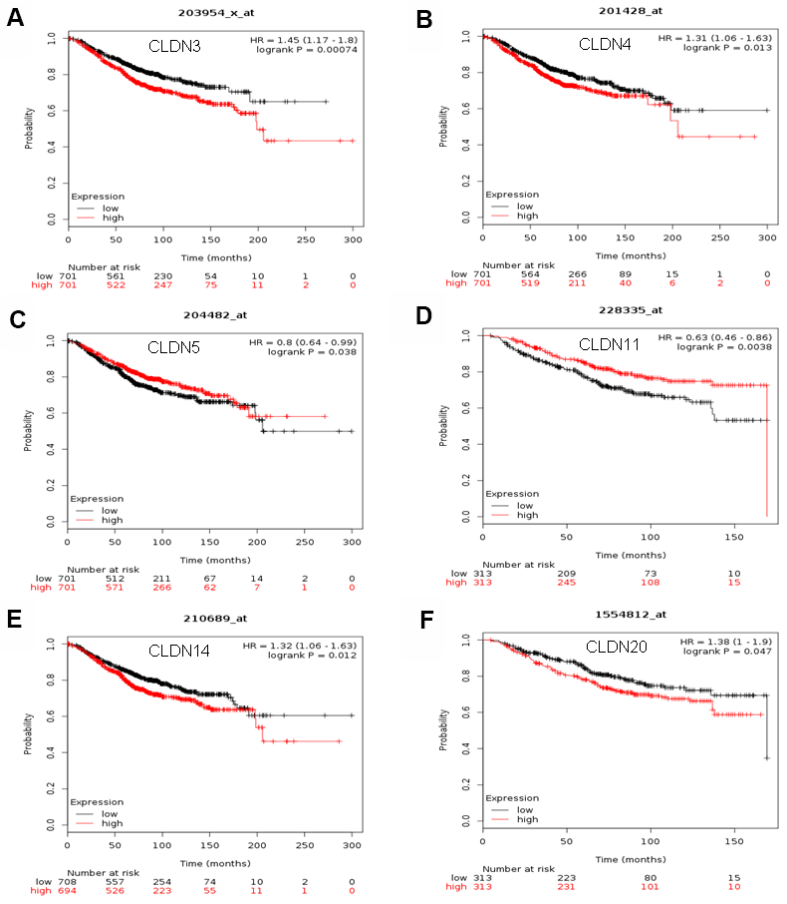
**The OS of mRNA levels of claudins in all patients with breast cancer (Kaplan–Meier plotter).** (**A**) CLDN3; (**B**) CLDN4; (**C**) CLDN5; (**D**) CLDN11; (**E**) CLDN14; (**F**) CLDN20. Abbreviations: OS, overall survival.

The Kaplan–Meier plotter was used to examine the prognostic values of claudin mRNA expression in all BC groups. Figure 5 shows all relapse-free survival (RFS) curves associated with the members of the claudin family. The results revealed that a high mRNA expression of CLDN2, 5, 6, 9, 10, 11, 14–20 was associated with better RFS (Figure 5A, 5C–5N). In contrast, a high mRNA expression of CLDN3 was associated with a poor prognosis of RFS (Figure 5B). In addition, we analyzed the relationship between mRNA expression of claudins and other prognostic indexes, including overall survival (OS), distant metastasis-free survival (DMFS), and post-progression survival (PPS). Figure 6 shows all OS curves associated with members of the claudin family. We found that a high mRNA expression of CLDN3, 4, 14, and 20 was associated with poor OS (Figure 6A, 6B, 6E, 6F), whereas a high mRNA expression of CLDN5 and CLDN11 was associated with better OS (Figure 6C, 6D). Similarly, a high mRNA expression of CLDN3 and CLDN7 was associated with a poor prognosis of DMFS and a high CLDN2 mRNA expression indicated better DMFS (Supplementary Table 6). In addition, a high mRNA expression of CLDN3, 4, and 14 was associated with poor PPS and a high mRNA expression of CLDN6 and CLDN18 indicated better PPS (Supplementary Table 6).

### Prognostic values of claudin mRNA expression in different molecular subtypes of breast cancer

We next analyzed the prognostic values of claudin mRNA expression in different molecular subtypes, including basal-like, luminal A, luminal B, and HER2+ (Supplementary Table 7).

In basal-like BC, a high mRNA expression of CLDN1 and CLDN7 correlated with poor RFS, whereas a high mRNA expression of CLDN6, 9, 10, 16, and 18 correlated with better RFS. In addition, a high mRNA expression of CLDN8 indicated better OS, whereas that of CLDN10 was associated with better DMFS. Moreover, a high mRNA expression of CLDN20 indicated poor PPS. The mRNA expression of other members of the claudin family members did not correlate with prognosis in basal-like BC.

In patients with luminal A BC, a high mRNA expression of CLDN1, 2, 5, 6, 8, 9, 10, 11, and 14 to 20 correlated with better RFS, whereas only a high CLDN3 expression was associated with poor RFS. In addition, a high mRNA expression of CLDN2 was associated with the poor OS but indicated better DMFS. Other members of the claudin family were not associated with any prognosis in patients with luminal A BC.

In patients with luminal B BC, a high mRNA expression of CLDN6, 8, 9, 10, 11, 15–18, 20, and 23 correlated with better RFS. In addition, a high expression of CLDN7 was associated with poor RFS and OS. No correlation with prognosis was found in the remaining members of the claudin family.

In patients with HER2+ BC, a high mRNA expression of CLDN9 and CLDN14 correlated with better RFS, whereas only a high CLDN8 expression was associated with poor RFS. In addition, a high expression of CLDN20 was associated with poor OS. No correlation with prognosis was found in the remaining members of the claudin family.

### Prognostic values of claudin mRNA expression in breast cancer with different clinicopathologic classifications

Next, we investigated the prognostic values of claudin mRNA expression in BC with different clinicopathologic classifications, including lymph node status and histologic grades (Supplementary Table 8). We found that a high mRNA expression of CLDN6, 9, 17, 18, and 20 correlated with poor PPS, whereas only high CLDN1 expression had better PPS in patients with lymph node positivity. In addition, a high CLDN11 expression was associated with better RFS in patients with lymph node positivity. A high CLDN8 expression resulted in poor DMFS, whereas a high CLDN20 expression was associated with poor OS in patients with lymph node positivity. In patients with lymph node negativity, a high CLDN3 expression correlated with the poor OS, DMFS, and PPS, whereas a high CLDN8 expression correlated with better DMFS. In addition, a high mRNA expression of CLDN9, 18, and 19 correlated with better PPS, whereas a high CLDN20 expression was associated with poor PPS. Moreover, a high CLDN9 expression indicated a better OS.

The second clinicopathologic classification we investigated was histologic grade (Supplementary Table 9). In patients with grade 1 BC, only high CLDN11 expression indicated better RFS, whereas high expression of CLDN3 and CLDN4 correlated with poor OS in patients with grade 2 BC, whereas a high CLDN12 expression had better OS. In addition, a high CLDN3 expression correlated with poor DMFS. A high expression of CLDN3, 4, 10, 16, and 18 correlated with poor PPS, whereas a high CLDN12 expression had better PPS. In patients with grade 3 BC, a high expression of CLDN1, 3, and 7 correlated with poor RFS, whereas a high CLDN16 expression was found to have better RFS. In addition, a high expression of CLDN1 and CLDN7 correlated with poor DMFS.

### Claudin gene alteration analysis

**Figure 7:**
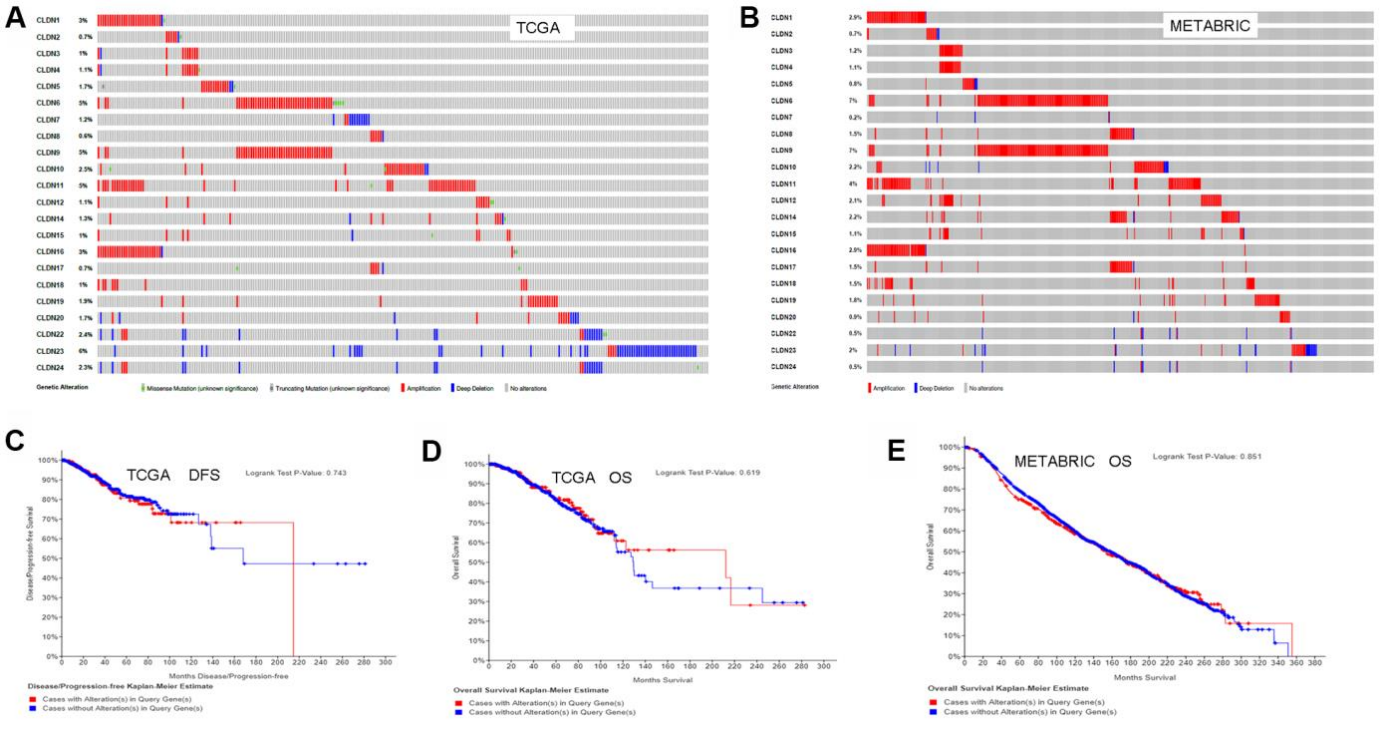
**Analysis of alterations in claudins in breast cancer (using cBioPortal for Cancer Genomics).** (**A**) OncoPrint of the TCGA dataset in cBioPortal. (**B**) OncoPrint of the METABRIC dataset in cBioPortal. (**C**) DFS analysis in cases with or without alterations in claudin genes of the TCGA dataset. (**D**) OS analysis in cases with or without alterations in claudin genes of the TCGA dataset. (**E**) OS analysis in cases with or without alterations in claudin genes of the METABRIC dataset. OncoPrint represents the distribution and proportion of samples with different kinds of alterations in the claudin family. Abbreviations: DFS, disease/progression-free survival; OS, overall survival.

We used the cBioPortal for Cancer Genomics database to analyze the alterations in the genes of the claudin family. As for the TCGA dataset (with 963 patients), 256 (27%) patients had altered claudin genes (Figure 7A). In addition, 485 (22%) patients had altered claudin genes as per the METABRIC dataset (Figure 7B). The genetic alterations included missense mutations, truncating mutations, amplifications, and deep deletions. However, the results from the two datasets showed no differences between OS/DFS and BC patients with or without alterations in claudin genes (Figure 7C–7E).

## DISCUSSION

We explored the mRNA expression, the correlation with clinicopathologic parameters, and prognostic values of 22 claudin genes in patients with BC. The majority of these genes had altered expression that could impact the survival of patients with BC. CLDN3, 5, and 11 could be used as promising therapeutic targets for BC. Compared with normal tissues, the expression of CLDN3, 4, and 7 was upregulated in BC tissues, a finding consistent with that of a previous study that reported elevated expression of these claudins in BC [22]. Although CLDN3 is known to function as a tumor suppressor in certain cancers [47–51], we found that a high CLDN3 expression was associated with poor RFS, OS, DMFS, and PPS in all patients. A high expression of CLDN3 and CLDN7 was associated with poor RFS in TNBC [23]. Moreover, CLDN3 expression correlated with SRB and NPI. In addition, CLDN3 expression correlated with almost all clinicopathologic parameters and impacted the prognosis in patients with lymph node negativity or grade 2 BC. These results indicated that CLDN3 could serve as a potential therapeutic intervention for BC.

Although a previous study associated upregulated CLDN5 expression in BC with poor RFS [27], we found that the low expression of CLDN5 and CLDN11 was associated with poor RFS and OS in all patients. Moreover, both of them correlated with SRB and with almost all clinicopathologic parameters. Similarly, another study found upregulated CLDN11 expression in BC [21]. However, the samples in these studies were small. Moreover, overexpressed CLDN5 reduced the paracellular permeability of hCMEC/D3 cells and decreased the invasion of lung adenocarcinoma A549 cells [52]. Insertion of *Claudin-5* gene in HECV cells substantially reduced the motility of the cells and their adhesiveness to the matrix, along with reduced angiogenic potential [53]. Downregulation of claudin-5 increased the permeability of the blood–tumor barrier [54, 55], suggesting it could prevent brain metastasis. Low CLDN11 expression has been used as a prognosis biomarker in certain cancers [56–58]. Moreover, miR-99b-induced downregulation of CLDN11 promoted metastasis of hepatocellular carcinoma [59]. Similarly, miR-421 promoted the proliferation and metastasis of gastric cancer cells by targeting claudin-11 [60]. In addition, low expression of CLDN5 and CLDN11 is associated with poor RFS in patients with luminal A BC. Based on these bioinformatics data, we speculate that the downregulated expression of CLDN5 and CLDN11 could be exploited to devise therapeutic strategies against BC.

Our study had some limitations. First, the expression or prognostic values of certain claudins in BC or its subtypes are still unknown due to limited samples. Second, mechanisms regulating the expression of claudins remain elusive. Based on the literature and functional modules available in online databases, we analyzed the methylation levels of claudins in BC. The results showed that CLDN4, 5, 6, 15, and 19 were hypermethylated in BC with lower mRNA expression compared to normal tissues. However, although CLDN16, 17, and 18 were hypermethylated, their mRNA expression did not differ. Interestingly, we found that CLDN11 was hypomethylated in BC with lower mRNA expression than in normal tissues. Methylation of CLDN11 promoter is known to be associated with the development and poor survival in several cancers [56–58]. Moreover, miRNA-induced reduced expression of CLDN11 promoted metastasis [59, 60], implying DNA methylation as one of the underlying regulatory mechanisms of at least certain claudins in BC. Third, this study showed that certain claudins functioned as tumor suppressors. Fourth, the results of bioinformatics analysis need to be validated by performing biological experiments.

In summary, we systematically analyzed the expression and prognostic value of claudins in BC. Our findings suggested that CLDN3, 5, and 11 could be used as promising therapeutic targets for BC.

## MATERIALS AND METHODS

### ONCOMINE analysis

ONCOMINE (http://www.oncomine.com), an online cancer microarray database, was used to analyze the mRNA levels of the claudin family in different cancers [61]. The search filters were set to the following: differential analysis (cancer vs. normal), cancer type (breast cancer), sample type (clinical specimen), data type (mRNA), and genes (CLDN1-12, CLDN14-20, and CLDN22-24). Thresholds were set as the following: gene rank, 10%; fold change, 2; and *p*-value, 0.05.

### UALCAN database

UALCAN (http://ualcan.path.uab.edu/index.html) is a portal for facilitating tumor subgroup gene expression and survival analyses [62]. It was used to evaluate the mRNA levels and promoter methylation levels of the claudin family in patients with BC and normal individuals. In addition, we evaluated the effects of the claudin family on the survival of patients with BC. The beta value indicates the level of DNA methylation ranging from 0 (unmethylated) to 1 (fully methylated). Different beta cut-off values have been considered to indicate hypermethylation (beta value: 0.7–0.5) or hypo-methylation (beta-value: 0.3–0.25). A *p*-value < 0.05 was considered significant.

### Breast cancer gene expression miner (bc-GenExMiner) v4.3

bc-GenExMiner v4.3 (http://bcgenex.centregauducheau.fr/BC-GEM/GEM-Accueil.php?js=1) [63], a statistical mining tool of published annotated BC transcriptomic data, was used to assess the correlation of expression of members of the claudin family with specific clinicopathologic features of BC, including age, nodal status, hormonal receptors status (ER and PR), HER2, pathologic subtype, NPI, and SBR grade. A *p*-value < 0.05 was considered significant.

### Kaplan–Meier plotter database

Kaplan–Meier plotter (https://kmplot.com/analysis/) [64], an online database established using gene expression data and survival information of 1,809 patients with BC downloaded from GEO, was used to analyze the prognostic values of members of the claudin family in all patients, in different molecular subtypes of BC, and different kinds of clinicopathologic classifications of BC. Only the best probe set of the claudin family was selected for this analysis. The hazard ratio (HR) with 95% confidence intervals and log-rank *p*-value were calculated and shown.

### The cancer genome atlas (TCGA) data and cBioPortal for cancer genomics database

The Cancer Genome Atlas consists of both sequencing and pathological data on 30 different cancers [65]. The cBioPortal for Cancer Genomics (http://www.cbioportal.org) contains large-scale cancer genomics datasets with functions such as visualization, downloading, and analysis [66]. We selected two BC datasets with most patients (TCGA, Provisional and METABRIC, Nature 2012 and Nat Commun, 2016) for further analysis using cBioPortal. The OncoPrint, OS, and DFS of the claudin family were analyzed online.

### Statistical analysis

Gene expression data from the Oncomine database were analyzed using the *p*-values, fold changes, and ranks. Gene expression and methylation levels from the UALCAN or bc-GenExMiner v4.3 database were compared using the *t*-test. Survival curves were generated using the Kaplan–Meier plots. The pairwise comparison in the SBR and NPI criteria was performed using Dunnett’s Tukey–Kramer test. *P*-values < 0.05 were considered significant. The details can be found on the webpage of the databases used.

## Supplementary Material

Supplementary Figure 1

Supplementary Table 1

Supplementary Table 2

Supplementary Table 3

Supplementary Table 4

Supplementary Table 5

Supplementary Table 6

Supplementary Table 7

Supplementary Table 8

Supplementary Table 9
