# Supplementary material for: Comprehensive analysis of expression and prognostic value of the claudin family in human breast cancer
Source: Aging (Albany NY). 2021 Mar 10;13(6):8777–96. doi: 10.18632/aging.202687 (PMC8034964; doi:10.18632/aging.202687)
Supplement: Supplementary Figure 1 [file aging-13-202687-s001.pdf]

SUPPLEMENTARY FIGURE

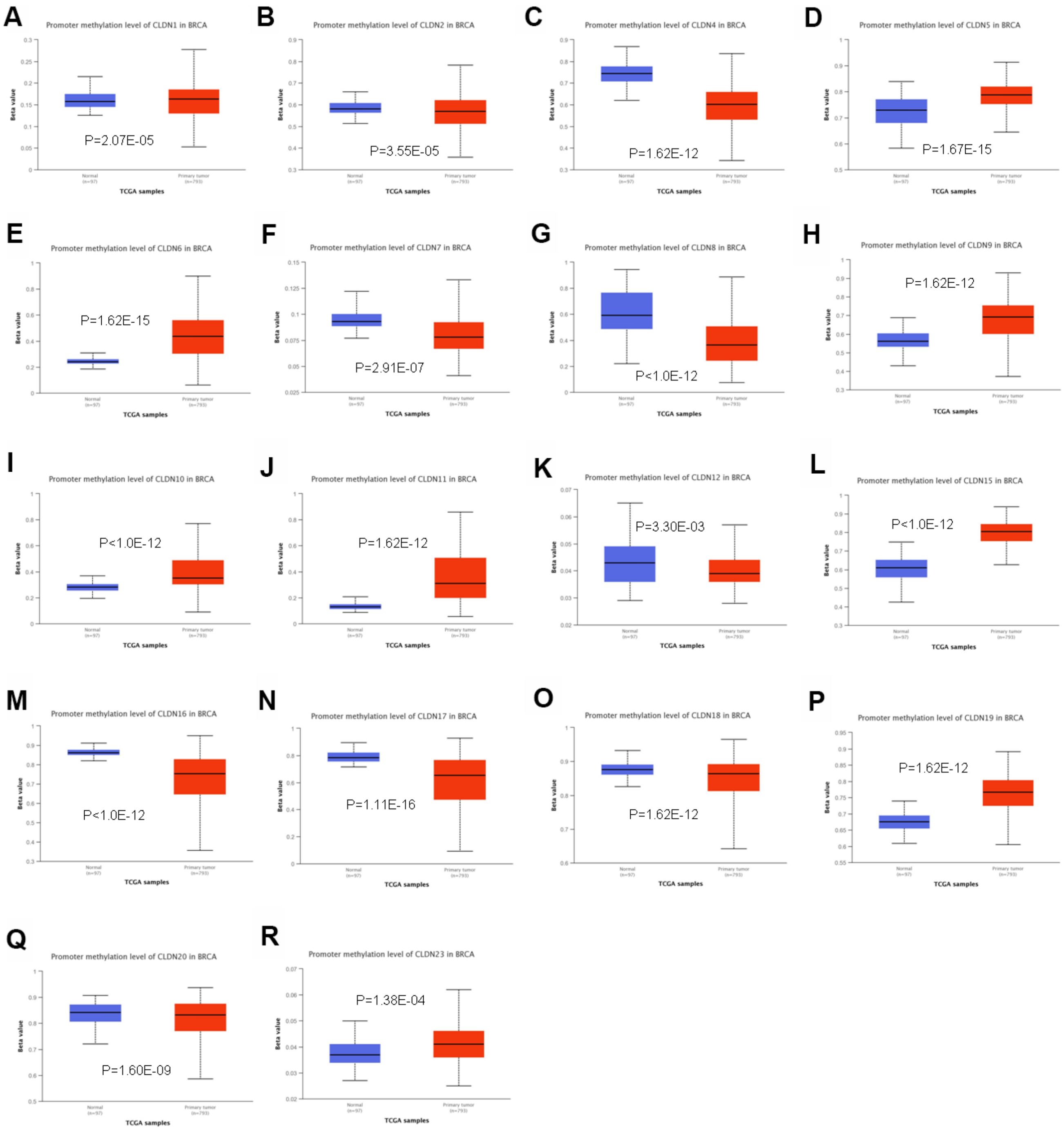

Supplementary Figure 1. Significant changes in claudin promoter methylation levels between breast cancer and normal tissues (UALCAN database). (**A**) CLDN1; (**B**) CLDN2; (**C**) CLDN4; (**D**) CLDN5; (**E**) CLDN6; (**F**) CLDN7; (**G**) CLDN8; (**H**) CLDN9; (**I**) CLDN10; (**J**) CLDN11; (**K**) CLDN12; (**L**) CLDN15; (**M**) CLDN16; (**N**) CLDN17; (**O**) CLDN18; (**P**) CLDN19; (**Q**) CLDN20; (**R**) CLDN23. The beta value indicates the level of DNA methylation ranging from 0 (unmethylated) to 1 (fully methylated). Different beta cut-off values have been considered to indicate hypermethylation (beta value: 0.7–0.5) or hypomethylation (beta-value: 0.3–0.25). A p-value < 0.05 was considered significant.
