# Supplementary material for: Comprehensive analysis of expression and prognostic value of the claudin family in human breast cancer
Source: Aging (Albany NY). 2021 Mar 10;13(6):8777–96. doi: 10.18632/aging.202687 (PMC8034964; doi:10.18632/aging.202687)
Supplement: Supplementary Table 1 [file aging-13-202687-s002.pdf]

Supplementary Table 1. Expression and functions of claudin in breast cancer.

Genes	Types of cancer	Expression level	Function role	References
CLDN1	breast cancer vs normal	down	NA	[14]
	recurrent group BC vs non-recurrent group	down	correlated with disease-free interval and lymph node metastasis	[15]
	TNBC	NA	associated with worse relapse-free survival (RFS)and overall survival	[16]
	basal-like breast cancer	up	involved in epithelial-mesenchymal-transition (EMT)	[17]
	breast cancer cells	NA	silencing CLDN1 inhibited epithelial to mesenchymal transition (EMT)	[18]
CLDN2	TNBC	NA	associated with the risk of recurrence in the lymph node positive subgroup	[16]
	breast cancer vs normal	down	associated with lymph node metastasis	[19]
	BC liver metastases compared to other metastatic sites	up	associated with metastasis-free interval	[20]
	breast cancer vs normal	down	NA	[21]
CLDN3	breast cancer vs normal	no difference	NA	[14]
	breast caner	up	NA	[22]
	TNBC	strong cytoplasmic claudin 3 expression	associated with survival.	[23]
CLDN4	breast cancer vs normal	no difference	NA	[14]
	recurrent group BC vs non-recurrent group	no significance	NA	[15]
	TNBC	NA	not associated with survival.	[16]
	breast caner	up	NA	[22]
	basal-like group BC as compared to not basal-like	up	NA	[24]
	breast caner	NA	correlated with tumour grade, Her2, ER and survival	[25]
	breast carcinoma in situ	up	claudin-4-low expression had a worse prognosis in carcinoma in situ	[26]
CLDN5	recurrent breast cancer	up	with lower relapse-free survival (RFS)	[27]
CLDN6	breast invasive ductal carcinomas	down	correlated with lymph node metastasis	[28]
	MCF-7/MDR cells	NA	High expression of CLDN6 confers chemoresistance on breast cancer	[29]
	TNBC cell line MDAMB231	NA	CLDN6 enhances chemoresistance to ADM	[30]
	breast cancer vs normal	down	NA	[21]
CLDN7	TNBC	NA	not associated with survival.	[16]
	breast caner	up	NA	[22]
	TNBC	strong cytoplasmic claudin 7 expression	associated with survival	[23]
CLDN11	breast cancer vs normal	up	NA	[21]
CLDN12	estrogen receptor (ER)-negative breast cancer	NA	associated with survival.	[31]
CLDN16	breast cancer vs normal	up	no significant association with overall survival	[32]
	node positive tumors compared to negative	down	associated with aggressive phenotype	[33]
	breast cancer vs normal	down	NA	[21]

Note: "up" means high expression; "down" means low expression; BC; breast cancer; TNBC, triple negative breast cancer; NA, not available.
