# Supplementary material for: Comprehensive analysis of expression and prognostic value of the claudin family in human breast cancer
Source: Aging (Albany NY). 2021 Mar 10;13(6):8777–96. doi: 10.18632/aging.202687 (PMC8034964; doi:10.18632/aging.202687)
Supplement: Supplementary Table 2 [file aging-13-202687-s003.doc]

**Supplementary Table 2. Datasets of the claudin family in breast cancer (ONCOMINE database).**

| **Gene** | **Types of cancer vs normal** | **Fold Change** | **t-Test** | **P-value** | **Dataset** |
| --- | --- | --- | --- | --- | --- |
| CLDN1 |  |  |  |  |  |
|  | Invasive Ductal Breast Carcinoma Stroma | 3.043 | 4.703 | 2.41E-04 | Ma Breast |
|  | Breast Phyllodes Tumor | -2.719 | -5.282 | 0.002 | Curtis Breast |
| CLDN2 |  |  |  |  |  |
|  | Invasive Lobular Breast Carcinoma | -4.357 | -2.418 | 0.019 | Radvanyi Breast |
|  | Invasive Lobular Breast Carcinoma | -2.283 | -2.155 | 0.039 | Turashvili Breast |
| CLDN3 |  |  |  |  |  |
|  | Intraductal Cribriform Breast Adenocarcinoma | 2.837 | 5.367 | 2.41E-06 | TCGA Breast |
|  | Lobular Breast Carcinoma | -2.219 | -3.844 | 0.006 | Perou Breast |
| CLDN4 |  |  |  |  |  |
|  | Mucinous Breast Carcinoma | 2.502 | 4.786 | 7.72E-06 | TCGA Breast |
|  | Lobular Breast Carcinoma | -2.329 | -2.39 | 0.032 | Perou Breast |
| CLDN5 |  |  |  |  |  |
|  | Invasive Breast Carcinoma Stroma | 8.083 | 13.554 | 2.29E-19 | Finak Breast |
|  | Medullary Breast Carcinoma | -6.637 | -21.011 | 5.14E-29 | Curtis Breast |
|  | Invasive Ductal and Invasive Lobular Breast Carcinoma | -4.467 | -19.145 | 5.17E-46 |  |
|  | Breast Carcinoma | -6.137 | -14.177 | 2.58E-11 |  |
|  | Tubular Breast Carcinoma | -4.638 | -18.046 | 3.20E-37 |  |
|  | Invasive Ductal Breast Carcinoma | -5.498 | -34.496 | 4.26E-78 |  |
|  | Invasive Breast Carcinoma | -5.522 | -11.378 | 1.32E-11 |  |
|  | Invasive Lobular Breast Carcinoma | -3.505 | -17.57 | 9.89E-48 |  |
|  | Mucinous Breast Carcinoma | -3.93 | -14.445 | 5.30E-24 |  |
|  | Breast Phyllodes Tumor | -2.134 | -3.885 | 7.00E-03 |  |
|  | Ductal Breast Carcinoma in Situ | -3.997 | -4.818 | 4.06E-04 |  |
|  | Mucinous Breast Carcinoma | -2.082 | -7.878 | 7.54E-06 | TCGA Breast |
|  | Mixed Lobular and Ductal Breast Carcinoma | -2.842 | -5.944 | 7.56E-05 |  |
|  | Invasive Ductal Breast Carcinoma | -3.334 | -13.859 | 2.56E-22 |  |
|  | Invasive Breast Carcinoma | -2.397 | -8.107 | 2.11E-13 |  |
|  | Invasive Lobular Breast Carcinoma | -2.078 | -6.246 | 7.14E-09 |  |
|  | Invasive Ductal Breast Carcinoma | -3.566 | -3.679 | 2.00E-03 | Radvanyi Breast |
|  | Invasive Breast Carcinoma | -3.609 | -8.155 | 1.00E-03 | Gluck Breast |
|  | Ductal Breast Carcinoma | -4.849 | -7.249 | 1.12E-04 | Richardson Breast |
| CLDN6 | NA |  |  |  |  |
| CLDN7 |  |  |  |  |  |
|  | Fibroadenoma | 2.236 | 7.583 | 8.85E-04 | Sorlie Breast |
|  | Intraductal Cribriform Breast Adenocarcinoma | 3.479 | 8.227 | 1.19E-06 | TCGA Breast |
|  | Mucinous Breast Carcinoma | 3.62 | 6.086 | 3.67E-04 |  |
|  | Invasive Ductal and Lobular Carcinoma | 2.051 | 5.063 | 6.50E-05 |  |
|  | Invasive Lobular Breast Carcinoma | 3.625 | 6.147 | 9.62E-09 |  |
|  | Male Breast Carcinoma | 2.496 | 4.519 | 4.00E-03 |  |
|  | Tubular Breast Carcinoma | 2.017 | 10.25 | 2.27E-20 | Curtis Breast |
|  | Invasive Breast Carcinoma | 2.007 | 4.9 | 2.06E-05 |  |
| CLDN8 |  |  |  |  |  |
|  | Invasive Lobular Breast Carcinoma | -2.796 | -3.638 | 2.00E-03 | Radvanyi Breast |
|  | Ductal Breast Carcinoma in Situ | -4.238 | -3.986 | 3.00E-03 |  |
|  | Invasive Lobular Breast Carcinoma | -5.484 | -3.697 | 1.00E-03 | Turashvili Breast |
|  | Invasive Ductal Breast Carcinoma | -9.446 | -3.089 | 1.10E-02 |  |
|  | Ductal Breast Carcinoma in Situ Stroma | -6.991 | -4.739 | 1.05E-04 | Ma Breast |
|  | Invasive Ductal Breast Carcinoma Stroma | -8.348 | -4.869 | 1.69E-04 |  |
|  | Invasive Ductal Breast Carcinoma Epithelia | -13.839 | -5.208 | 2.13E-04 |  |
|  | Ductal Breast Carcinoma in Situ Epithelia | -6.23 | -3.552 | 3.00E-03 |  |
|  | Medullary Breast Carcinoma | -3.27 | -11.277 | 1.34E-19 | Curtis Breast |
|  | Mucinous Breast Carcinoma | -2.878 | -9.993 | 4.64E-18 |  |
|  | Breast Carcinoma | -2.975 | -5.995 | 5.26E-06 |  |
|  | Invasive Lobular Breast Carcinoma | -2.382 | -9.894 | 5.08E-20 |  |
|  | Tubular Breast Carcinoma | -2.416 | -8.975 | 1.29E-16 |  |
|  | Invasive Ductal and Invasive Lobular Breast Carcinoma | -2.445 | -8.978 | 5.57E-17 |  |
|  | Invasive Ductal Breast Carcinoma Stroma | -5.043 | -3.492 | 1.00E-03 | Karnoub Breast |
|  | Ductal Breast Carcinoma | -15.33 | -7.477 | 1.48E-06 | Richardson Breast |
|  | Mucinous Breast Carcinoma | -6.893 | -4.631 | 0.003 | TCGA Breast |
|  | Male Breast Carcinoma | -24.488 | -6.543 | 0.003 |  |
| CLDN9 |  |  |  |  |  |
|  | Invasive Breast Carcinoma Stroma | 3.269 | 12.845 | 1.03E-14 | Finak Breast |
| CLDN10 |  |  |  |  |  |
|  | Lobular Breast Carcinoma | -3.708 | -3.325 | 0.011 | Sorlie Breast |
|  | Fibroadenoma | -2.117 | -5.306 | 0.036 |  |
|  | Ductal Breast Carcinoma | -2.434 | -4.762 | 0.004 | Sorlie Breast 2001 |
|  | Mixed Lobular and Ductal Breast Carcinoma | -5.172 | -8.195 | 2.44E-06 | TCGA Breast |
|  | Intraductal Cribriform Breast Adenocarcinoma | -7.292 | -10.369 | 2.42E-04 |  |
|  | Invasive Breast Carcinoma | -3.459 | -8.261 | 6.10E-14 |  |
|  | Invasive Lobular Breast Carcinoma | -3.047 | -6.468 | 4.99E-09 |  |
|  | Invasive Ductal Breast Carcinoma | -3.969 | -11.268 | 2.00E-20 |  |
|  | Invasive Ductal and Lobular Carcinoma | -2.44 | -5.313 | 4.00E-03 |  |
| CLDN11 |  |  |  |  |  |
|  | Ductal Breast Carcinoma | -13.276 | -13.342 | 3.49E-17 | Richardson Breast |
|  | Invasive Ductal Breast Carcinoma | -2.952 | -3.777 | 0.000572 | Radvanyi Breast |
|  | Intraductal Cribriform Breast Adenocarcinoma | -4.947 | -12.626 | 1.11E-07 | TCGA Breast |
|  | Invasive Breast Carcinoma | -2.721 | -10.874 | 7.57E-20 |  |
|  | Invasive Lobular Breast Carcinoma | -3.752 | -8.451 | 1E-12 |  |
|  | Mucinous Breast Carcinoma | -3.92 | -8.004 | 0.000407 |  |
|  | Invasive Ductal Breast Carcinoma | -5.179 | -16.611 | 1.52E-26 |  |
|  | Medullary Breast Carcinoma | -4.94 | -15.537 | 1.24E-20 | Curtis Breast |
|  | Breast Carcinoma | -4.413 | -10.249 | 9.32E-09 |  |
|  | Invasive Ductal Breast Carcinoma | -3.674 | -26.982 | 6.79E-64 |  |
|  | Invasive Ductal and Invasive Lobular Breast Carcinoma | -2.615 | -11.976 | 9.96E-25 |  |
|  | Invasive Breast Carcinoma | -3.467 | -6.87 | 2.73E-07 |  |
|  | Invasive Lobular Breast Carcinoma | -2.393 | -11.827 | 1.01E-26 |  |
|  | Mucinous Breast Carcinoma | -3.609 | -10.758 | 4.49E-16 |  |
|  | Tubular Breast Carcinoma | -2.382 | -9.855 | 2.32E-17 |  |
|  | Lobular Breast Carcinoma | -4.162 | -6.257 | 0.004 | Zhao Breast |
|  | nvasive Ductal Breast Carcinoma | -5.436 | -7.44 | 0.002 |  |
|  | Invasive Ductal Breast Carcinoma Stroma | -2.475 | -3.251 | 0.003 | Karnoub Breast |
| CLDN12 |  |  |  |  |  |
|  | Intraductal Cribriform Breast Adenocarcinoma | 2.245 | 6.171 | 0.003 | TCGA Breast |
|  | Invasive Lobular Breast Carcinoma | -3.212 | -2.795 | 0.015 | Turashvili Breast |
| CLDN14 |  |  |  |  |  |
|  | Invasive Breast Carcinoma | 2.006 | 13.613 | 1.55E-18 | Gluck Breast |
|  | Invasive Breast Carcinoma | 4.034 | 10.535 | 1.36E-19 | TCGA Breast |
|  | Mixed Lobular and Ductal Breast Carcinoma | 2.161 | 4.953 | 1.16E-04 |  |
|  | Invasive Ductal Breast Carcinoma | 3.192 | 13.181 | 4.88E-24 |  |
|  | Invasive Lobular Breast Carcinoma | 3.035 | 6.501 | 7.45E-09 |  |
|  | Intraductal Cribriform Breast Adenocarcinoma | 4.673 | 6.853 | 4.00E-03 |  |
|  | Invasive Ductal Breast Carcinoma | -2.331 | -2.322 | 3.40E-02 | Turashvili Breast |
| CLDN15 | NA |  |  |  |  |
| CLDN16 |  |  |  |  |  |
|  | Intraductal Cribriform Breast Adenocarcinoma | -2.053 | -5.814 | 0.001 | TCGA Breast |
| CLDN17 | NA |  |  |  |  |
| CLDN18 |  |  |  |  |  |
|  | Invasive Ductal Breast Carcinoma | -2.301 | -4.232 | 1.89E-04 | Turashvili Breast |
| CLDN19 |  |  |  |  |  |
|  | Mucinous Breast Carcinoma | -10.49 | -14.668 | 6.00E-11 | TCGA Breast |
|  | Mixed Lobular and Ductal Breast Carcinoma | -3.652 | -7.307 | 2.08E-06 |  |
|  | Male Breast Carcinoma | -4.504 | -10.7 | 5.63E-07 |  |
|  | Invasive Breast Carcinoma | -5.289 | -10.349 | 7.87E-19 |  |
|  | Invasive Ductal Breast Carcinoma | -6.98 | -15.032 | 1.71E-24 |  |
|  | Invasive Lobular Breast Carcinoma | -4.181 | -6.656 | 2.98E-09 |  |
| CLDN20 | NA |  |  |  |  |
| CLDN22 |  |  |  |  |  |
|  | Invasive Ductal and Lobular Carcinoma | -2.229 | -11.529 | 5.33E-06 | TCGA Breast |
| CLDN23 |  |  |  |  |  |
|  | Invasive Lobular Breast Carcinoma | -3.776 | -2.435 | 0.025 | Turashvili Breast |
| CLDN24 | NA |  |  |  |  |
| **Note**：NA,not avaliable. | | | | | |
|  |  |  |  |  |  |
