# Supplementary material for: Comprehensive analysis of expression and prognostic value of the claudin family in human breast cancer
Source: Aging (Albany NY). 2021 Mar 10;13(6):8777–96. doi: 10.18632/aging.202687 (PMC8034964; doi:10.18632/aging.202687)
Supplement: Supplementary Table 3 [file aging-13-202687-s004.doc]

**Supplementary Table 3. The expression of the claudin family of breast cancer (UALCAN database).**

| **Parameters** | **CLDN1** | | **CLDN2** | | **CLDN3** | | **CLDN4** | | **CLDN5** | | **CLDN6** | |
| --- | --- | --- | --- | --- | --- | --- | --- | --- | --- | --- | --- | --- |
|  | **mRNA** | **p-value** | **mRNA** | **p-value** | **mRNA** | **p-value** | **mRNA** | **p-value** | **mRNA** | **p-value** | **mRNA** | **p-value** |
| **Expression** |  |  |  |  |  |  |  |  |  |  |  |  |
| Normal | - | 0.650 | - | 0.556 | sig | <1.0E-12 | sig | 2.9E-8 | sig | <1.0E-12 | sig | 5.62E-04 |
| Primary tumor | - |  | - |  | high |  | high |  | low |  | high |  |
| **DNA methylation** |  |  |  |  |  |  |  |  |  |  |  |  |
| Normal | sig | 2.07E-05 | sig | 3.55E-05 | - | 0.640 | sig | 1.62E-12 | sig | 1.67E-15 | sig | 1.62E-12 |
| Primary tumor | high |  | low |  | - |  | low |  | high |  | high |  |
| **Parameters** | **CLDN7** | | **CLDN8** | | **CLDN9** | | **CLDN10** | | **CLDN11** | | **CLDN12** | |
|  | **mRNA** | **p-value** | **mRNA** | **p-value** | **mRNA** | **p-value** | **mRNA** | **p-value** | **mRNA** | **p-value** | **mRNA** | **p-value** |
| **Expression** |  |  |  |  |  |  |  |  |  |  |  |  |
| Normal | sig | 1.62E-12 | sig | <1.0E-12 | sig | 1.21E-14 | - | 0.916 | sig | 1.62E-12 | - | 0.545 |
| Primary tumor | high |  | low |  | high |  | - |  | low |  | - |  |
| **DNA methylation** |  |  |  |  |  |  |  |  |  |  |  |  |
| Normal | sig | 2.91E-07 | sig | <1.0E-12 | sig | 1.62E-12 | sig | <1.0E-12 | sig | 1.62E-12 | sig | 3.30E-03 |
| Primary tumor | low |  | low |  | high |  | high |  | high |  | low |  |
| **Parameters** | **CLDN14** | | **CLDN15** | | **CLDN16** | | **CLDN17** | | **CLDN18** | | **CLDN19** | |
|  | **mRNA** | **p-value** | **mRNA** | **p-value** | **mRNA** | **p-value** | **mRNA** | **p-value** | **mRNA** | **p-value** | **mRNA** | **p-value** |
| **Expression** |  |  |  |  |  |  |  |  |  |  |  |  |
| Normal | sig | <1.0E-12 | sig | 2.33E-10 | - | 0.614 | - | 0.101 | - | 0.980 | sig | 1.62E-12 |
| Primary tumor | high |  | low |  | - |  | - |  | - |  | low |  |
| **DNA methylation** |  |  |  |  |  |  |  |  |  |  |  |  |
| Normal | - | 0.143 | sig | <1.0E-12 | sig | <1.0E-12 | sig | 1.11E-16 | sig | 1.62E-12 | sig | 1.62E-12 |
| Primary tumor | - |  | high |  | low |  | low |  | low |  | high |  |
| **Parameters** | **CLDN20** | | **CLDN22** | | **CLDN23** | | **CLDN24** | |  |  |  |  |
|  | **mRNA** | **p-value** | **mRNA** | **p-value** | **mRNA** | **p-value** | **mRNA** | **p-value** |  |  |  |  |
| **Expression** |  |  |  |  |  |  |  |  |  |  |  |  |
| Normal | sig | 1.70E-06 | NA |  | - | 0.551 | NA |  |  |  |  |  |
| Primary tumor | low |  |  |  | - |  |  |  |  |  |  |  |
| **DNA methylation** |  |  |  |  |  |  |  |  |  |  |  |  |
| Normal | sig | 1.60E-09 | NA |  | sig | 1.38E-04 | NA |  |  |  |  |  |
| Primary tumor | low |  |  |  | high |  |  |  |  |  |  |  |
| **Note:**“high” means high expression; “low” means low expression. | | | | | | | | | | | | |
|  |  |  |  |  |  |  |  |  |  |  |  |  |
|  |  |  |  |  |  |  |  |  |  |  |  |  |
