# Supplementary material for: Comprehensive analysis of expression and prognostic value of the claudin family in human breast cancer
Source: Aging (Albany NY). 2021 Mar 10;13(6):8777–96. doi: 10.18632/aging.202687 (PMC8034964; doi:10.18632/aging.202687)
Supplement: Supplementary Table 4 [file aging-13-202687-s005.doc]

**Supplementary Table 4. Relationship between the claudin family and the clinicopathologic parameters of breast cancer (bc-GenExMiner v4.3).**

| **Parameters** | **CLDN1** | | **CLDN2** | | **CLDN3** | | **CLDN4** | | **CLDN5** | | **CLDN6** | | **CLDN7** | | **CLDN8** | | **CLDN9** | | **CLDN10** | | **CLDN11** | |
| --- | --- | --- | --- | --- | --- | --- | --- | --- | --- | --- | --- | --- | --- | --- | --- | --- | --- | --- | --- | --- | --- | --- |
|  | **mRNA** | **p-value** | **mRNA** | **p-value** | **mRNA** | **p-value** | **mRNA** | **p-value** | **mRNA** | **p-value** | **mRNA** | **p-value** | **mRNA** | **p-value** | **mRNA** | **p-value** | **mRNA** | **p-value** | **mRNA** | **p-value** | **mRNA** | **p-value** |
| Age (years) |  |  |  |  |  |  |  |  |  |  |  |  |  |  |  |  |  |  |  |  |  |  |
| <51 | - | 0.4625 | sig | 0.0394 | sig | 0.0044 | - | 0.1405 | - | 0.5489 | - | 0.5362 | - | 0.2876 | sig | 0.0002 | - | 0.1005 | sig | p<0.0001 | sig | p<0.0001 |
| >51 | - |  | low |  | high |  | - |  | - |  | - |  | - |  | low |  | - |  | low |  | low |  |
| Nodal status |  |  |  |  |  |  |  |  |  |  |  |  |  |  |  |  |  |  |  |  |  |  |
| Negative | - | 0.2511 | sig | p<0.0001 | sig | p<0.0001 | sig | p<0.0001 | - | 0.3916 | - | 0.2272 | sig | p<0.0001 | - | 0.5567 | - | 0.2028 | sig | 0.0372 | - | 0.3526 |
| Postive | - |  | low |  | high |  | high |  | - |  | - |  | high |  | - |  | - |  | low |  | - |  |
| ER (IHC) |  |  |  |  |  |  |  |  |  |  |  |  |  |  |  |  |  |  |  |  |  |  |
| Negative | sig | p<0.0001 | - | 0.3477 | sig | 0.0203 | sig | p<0.0001 | sig | p<0.0001 | sig | p<0.0001 | sig | p<0.0001 | sig | p<0.0001 | sig | p<0.0001 | sig | p<0.0001 | sig | p<0.0001 |
| Postive | low |  | - |  | high |  | low |  | high |  | low |  | high |  | low |  | low |  | low |  | high |  |
| PR (IHC) |  |  |  |  |  |  |  |  |  |  |  |  |  |  |  |  |  |  |  |  |  |  |
| Negative | sig | p<0.0001 | sig | 0.0378 | - | 0.3144 | sig | p<0.0001 | sig | p<0.0001 | sig | p<0.0001 | sig | p<0.0001 | sig | p<0.0001 | sig | 0.0231 | sig | p<0.0001 | sig | p<0.0001 |
| Postive | low |  | low |  | - |  | low |  | high |  | low |  | high |  | low |  | low |  | low |  | high |  |
| HER2 (IHC) |  |  |  |  |  |  |  |  |  |  |  |  |  |  |  |  |  |  |  |  |  |  |
| Negative | - | 0.3027 | sig | 0.0021 | - | 0.1928 | sig | 0.0052 | sig | 0.0392 | - | 0.7519 | - | 0.9971 | - | 0.2008 | - | 0.2520 | - | 0.2221 | - | 0.8659 |
| Postive | - |  | high |  | - |  | high |  | low |  | - |  | - |  | - |  | - |  | - |  | - |  |
| Triple-negtive status |  |  |  |  |  |  |  |  |  |  |  |  |  |  |  |  |  |  |  |  |  |  |
| Not | sig | p<0.0001 | - | 0.3752 | sig | 0.0036 | - | 0.6682 | sig | p<0.0001 | sig | p<0.0001 | sig | p<0.0001 | sig | p<0.0001 | sig | 0.0024 | sig | p<0.0001 | sig | p<0.0001 |
| TNBC | high |  | - |  | low |  | - |  | low |  | high |  | low |  | high |  | high |  | high |  | low |  |
| Basal-like stasus |  |  |  |  |  |  |  |  |  |  |  |  |  |  |  |  |  |  |  |  |  |  |
| Not | sig | p<0.0001 | sig | 0.0207 | sig | 0.0004 | sig | p<0.0001 | sig | p<0.0001 | sig | p<0.0001 | sig | 0.0004 | sig | p<0.0001 | sig | p<0.0001 | sig | p<0.0001 | sig | p<0.0001 |
| Basal-like | high |  | high |  | low |  | high |  | low |  | high |  | low |  | high |  | high |  | high |  | low |  |
| **Parameters** | **CLDN12** | | **CLDN14** | | **CLDN15** | | **CLDN16** | | **CLDN17** | | **CLDN18** | | **CLDN19** | | **CLDN20** | | **CLDN22** | | **CLDN23** | | **CLDN24** | |
|  | **mRNA** | **p-value** | **mRNA** | **p-value** | **mRNA** | **p-value** | **mRNA** | **p-value** | **mRNA** | **p-value** | **mRNA** | **p-value** | **mRNA** | **p-value** | **mRNA** | **p-value** | **mRNA** | **p-value** | **mRNA** | **p-value** | **mRNA** | **p-value** |
| Age (years) |  |  |  |  |  |  |  |  |  |  |  |  |  |  |  |  |  |  |  |  |  |  |
| <51 | - | 0.3270 | - | 0.1030 | - | 0.1954 | - | 0.6365 | - | 0.6353 | - | 0.8845 | sig | 0.0169 | - | 0.1391 | - | 0.8305 | sig | p<0.0001 | - | 0.4403 |
| >51 | - |  | - |  | - |  | - |  | - |  | - |  | low |  | - |  | - |  | low |  | - |  |
| Nodal status |  |  |  |  |  |  |  |  |  |  |  |  |  |  |  |  |  |  |  |  |  |  |
| Negative | - | 0.1347 | - | 0.0770 | sig | p<0.0001 | - | 0.3031 | - | 0.2974 | - | 0.7703 | sig | p<0.0001 | - | 0.5185 | - | 0.5187 | sig | 0.0219 | - | 0.0731 |
| Postive | - |  | - |  | high |  | - |  | - |  | - |  | low |  | - |  | - |  | low |  | - |  |
| ER (IHC) |  |  |  |  |  |  |  |  |  |  |  |  |  |  |  |  |  |  |  |  |  |  |
| Negative | sig | p<0.0001 | sig | 0.0008 | - | 0.3919 | sig | p<0.0001 | sig | 0.0175 | - | 0.1727 | - | 0.2717 | - | 0.1714 | - | 0.2465 | sig | p<0.0001 | sig | 0.0469 |
| Postive | high |  | low |  | - |  | low |  | low |  | - |  | - |  | - |  | - |  | low |  | low |  |
| PR (IHC) |  |  |  |  |  |  |  |  |  |  |  |  |  |  |  |  |  |  |  |  |  |  |
| Negative | sig | p<0.0001 | sig | 0.0018 | - | 0.4125 | sig | 0.0056 | - | 0.9336 | - | 0.3483 | - | 0.2160 | - | 0.1215 | sig | 0.0104 | sig | p<0.0001 | sig | 0.0254 |
| Postive | high |  | low |  | - |  | low |  | - |  | - |  | - |  | - |  | low |  | low |  | low |  |
| HER2 (IHC) |  |  |  |  |  |  |  |  |  |  |  |  |  |  |  |  |  |  |  |  |  |  |
| Negative | sig | p<0.0001 | - | 0.0771 | - | 0.6903 | - | 0.0981 | - | 0.6518 | - | 0.4619 | - | 0.1268 | - | 0.6706 | - | 0.9876 | - | 0.2144 | - | 0.4370 |
| Postive | low |  | - |  | - |  | - |  | - |  | - |  | - |  | - |  | - |  | - |  | - |  |
| Triple-negtive status |  |  |  |  |  |  |  |  |  |  |  |  |  |  |  |  |  |  |  |  |  |  |
| Not | sig | p<0.0001 | - | 0.0857 | - | 0.9696 | sig | p<0.0001 | - | 0.5144 | - | 0.7404 | sig | 0.0123 | sig | p<0.0001 | - | 0.1588 | sig | p<0.0001 | - | 0.1096 |
| TNBC | low |  | - |  | - |  | high |  | - |  | - |  | low |  | high |  | - |  | high |  | - |  |
| Basal-like stasus |  |  |  |  |  |  |  |  |  |  |  |  |  |  |  |  |  |  |  |  |  |  |
| Not | sig | p<0.0001 | sig | 0.0033 | - | 0.3919 | sig | p<0.0001 | - | 0.1993 | - | 0.9479 | sig | 0.0057 | - | 0.4828 | sig | 0.0436 | sig | p<0.0001 | - | 0.0896 |
| Basal-like | low |  | high |  | - |  | high |  | - |  | - |  | low |  | - |  | high |  | high |  | - |  |
| **Notes:** All the data of the claudins family were based on bc-GenExMiner v4.3. “sig” means the comparson between two groups was considered statistically significant . P<0.05 was considered statistically significant. “high” means high expression; “low” means low expression. **Abbreviations:** bc-GenExMiner, Breast cancer Gene-Expression Miner; ER, estrogen receptor; HER2, human epidermal growth factor receptor 2; IHC, immunohistochemistry; PR, progesterone receptor; TNBC, triple-negative breast cancer. | | | | | | | | | | | | | | | | | | | | | | |
|  |  |  |  |  |  |  |  |  |  |  |  |  |  |  |  |  |  |  |  |  |  |  |
|  |  |  |  |  |  |  |  |  |  |  |  |  |  |  |  |  |  |  |  |  |  |  |
|  |  |  |  |  |  |  |  |  |  |  |  |  |  |  |  |  |  |  |  |  |  |  |
|  |  |  |  |  |  |  |  |  |  |  |  |  |  |  |  |  |  |  |  |  |  |  |
|  |  |  |  |  |  |  |  |  |  |  |  |  |  |  |  |  |  |  |  |  |  |  |
