# Supplementary material for: Comprehensive analysis of expression and prognostic value of the claudin family in human breast cancer
Source: Aging (Albany NY). 2021 Mar 10;13(6):8777–96. doi: 10.18632/aging.202687 (PMC8034964; doi:10.18632/aging.202687)
Supplement: Supplementary Table 5 [file aging-13-202687-s006.pdf]

SUPPLEMENTARY TABLE

Supplementary Table 5. Results of Dunnett's Tukey–Kramer test for pairwise comparison in scarff bloom and richardson and nottingham prognostic index criteria.

Gene	Pairwise comparison of SBR	P-value	Pairwise comparison of NPI	P-value
CLDN1	No significant	0.0553	NPI2 > NPI1	< 0.01
			NPI3 = NPI1	> 0.10
			NPI3 = NPI2	> 0.10
CLDN2	No significant	0.568	No significant	0.2713
CLDN3	SBR2 > SBR1	<0.0001	NPI2 > NPI1	<0.0001
	SBR3 > SBR1	<0.0001	NPI3 > NPI1	<0.0001
	SBR3 > SBR2	<0.0001	NPI3 > NPI2	<0.0001
CLDN4	SBR3 > SBR1	<0.0001	NPI2 > NPI1	<0.0001
	SBR3 > SBR2	<0.0001	NPI3 > NPI1	<0.0001
	SBR2 > SBR1	<0.0001	NPI3 = NPI2	>0.1
CLDN5	SBR2 < SBR1	<0.0001	NPI2 < NPI1	<0.0001
	SBR3 < SBR1	<0.0001	NPI3 < NPI1	<0.0001
	SBR3 < SBR2	<0.0001	NPI3 = NPI2	>0.1
CLDN6	SBR3 > SBR1	<0.01	NPI2 > NPI1	<0.05
	SBR3 > SBR2	<0.01	NPI3 = NPI1	>0.1
	SBR2 = SBR1	>0.1	NPI3 = NPI2	>0.1
CLDN7	SBR2 > SBR1	<0.05	NPI2 > NPI1	<0.05
	SBR3 < SBR2	<0.1	NPI3 > NPI1	<0.05
	SBR3 = SBR1	>0.1	NPI3 = NPI2	>0.1
CLDN8	No significant	0.3213	No significant	0.1274
CLDN9	SBR3 > SBR1	<0.0001	NPI2 > NPI1	<0.0001
	SBR3 > SBR2	<0.0001	NPI3 > NPI1	<0.0001
	SBR2 = SBR1	>0.1	NPI3 > NPI2	<0.01
CLDN10	SBR3 > SBR1	<0.0001	NPI2 > NPI1	<0.0001
	SBR3 > SBR2	<0.0001	NPI3 > NPI1	<0.001
	SBR2 = SBR1	>0.1	NPI3 = NPI2	>0.1
CLDN11	SBR2 < SBR1	<0.0001	NPI2 < NPI1	<0.0001
	SBR3 < SBR1	<0.0001	NPI3 < NPI1	<0.01
	SBR3 < SBR2	<0.0001	NPI3 = NPI2	>0.1
CLDN12	SBR3 < SBR1	<0.0001	NPI2 < NPI1	<0.01
	SBR3 < SBR2	<0.0001	NPI3 < NPI1	<0.01
	SBR2 < SBR1	<0.1	NPI3 = NPI2	>0.1
CLDN14	SBR3 > SBR1	<0.01	No significant	0.1355
	SBR3 > SBR2	<0.05		
	SBR2 = SBR1	>0.1		
CLDN15	SBR3 < SBR1	<0.05	No significant	0.0732
	SBR3 < SBR2	<0.05		
	SBR2 = SBR1	>0.1		
CLDN16	SBR3 > SBR2	<0.0001	No significant	0.6659
	SBR3 > SBR1	<0.05		
	SBR2 = SBR1	>0.1		
CLDN17	SBR3 > SBR2	<0.05	No significant	0.6285
	SBR2 = SBR1	>0.1		
	SBR3 = SBR1	>0.1		
CLDN18	No significant	0.5717	No significant	0.9423
CLDN19	No significant	0.5957	No significant	0.1165
CLDN20	No significant	0.0907	No significant	0.8386

CLDN22	No significant	0.8599	No significant	0.7234
CLDN23	SBR3 > SBR1	<0.0001	NPI2 > NPI1	<0.0001
	SBR3 > SBR2	<0.0001	NPI3 > NPI1	<0.0001
	SBR2 = SBR1	>0.1	NPI3 = NPI2	>0.01
CLDN24	SBR2 > SBR1	<0.1	No significant	0.7341
	SBR3 > SBR1	<0.1		
	SBR3 = SBR2	>0.1		
