# Supplementary material for: Comprehensive analysis of expression and prognostic value of the claudin family in human breast cancer
Source: Aging (Albany NY). 2021 Mar 10;13(6):8777–96. doi: 10.18632/aging.202687 (PMC8034964; doi:10.18632/aging.202687)
Supplement: Supplementary Table 6 [file aging-13-202687-s007.doc]

**Supplementary Table 6. Survival analyses of the claudin family in all patients with breast cancer (Kaplan–Meier plotter).**

| **Parameters** | **CLDN1** | | **CLDN2** | | **CLDN3** | | **CLDN4** | | **CLDN5** | | **CLDN6** | |
| --- | --- | --- | --- | --- | --- | --- | --- | --- | --- | --- | --- | --- |
|  | **HR(95%CI)** | **p-value** | **HR(95%CI)** | **p-value** | **HR(95%CI)** | **p-value** | **HR(95%CI)** | **p-value** | **HR(95%CI)** | **p-value** | **HR(95%CI)** | **p-value** |
| RFS | 1.08(0.92-1.26) | 0.34 | 0.75(0.64-0.88) | 0.00034 | 1.29(1.16-1.44) | 4.60E-06 | 1.08(0.97-1.2) | 0.17 | 0.78(0.7-0.87) | 9.40E-06 | 0.7(0.63-0.79) | 2.80E-10 |
| OS | 1.1(0.81-1.51) | 0.54 | 0.89(0.65-1.22) | 0.49 | 1.45(1.17-1.8) | 7.40E-04 | 1.31(1.06-1.63) | 0.013 | 0.8(0.64-0.99) | 3.80E-02 | 0.89(0.72-1.1) | 0.27 |
| DMFS | 1.18(0.86-1.64) | 0.31 | 0.79(0.57-1.09) | 0.15 | 1.37(1.13-1.66) | 0.0015 | 1.18(0.97-1.43) | 0.093 | 0.86(0.71-1.04) | 0.12 | 0.95(0.78-1.15) | 0.58 |
| PPS | 0.88(0.61-1.25) | 0.48 | 0.91(0.64-1.3) | 0.6 | 1.38(1.08-1.76) | 0.0099 | 1.37(1.07-0.011) | 0.011 | 0.93(0.73-1.19) | 0.57 | 0.85(0.66-1.08) | 0.18 |
| **Parameters** | **CLDN7** | | **CLDN8** | | **CLDN9** | | **CLDN10** | | **CLDN11** | | **CLDN12** | |
|  | **HR(95%CI)** | **p-value** | **HR(95%CI)** | **p-value** | **HR(95%CI)** | **p-value** | **HR(95%CI)** | **p-value** | **HR(95%CI)** | **p-value** | **HR(95%CI)** | **p-value** |
| RFS | 1.09(0.98-1.22) | 0.1 | 0.93(0.83-1.04) | 0.19 | 0.73(0.65-0.81) | 1.20E-08 | 0.77(0.69-0.86) | 2.10E-06 | 0.71(0.63-0.79) | 5.70E-10 | 0.88(0.75-1.02) | 0.092 |
| OS | 1.21(0.98-1.5) | 0.082 | 0.94(0.76-1.17) | 0.58 | 0.92(0.74-1.14) | 0.45 | 1.03(0.83-1.27) | 0.81 | 0.63(0.46-0.86) | 0.0038 | 0.77(0.56-1.05) | 0.096 |
| DMFS | 1.32(1.09-1.61) | 0.0045 | 0.82(0.68-1) | 0.045 | 1.08(0.89-1.32) | 0.41 | 0.97(0.8-1.17) | 0.73 | 0.73(0.53-1.01) | 0.055 | 0.92(0.67-1.27) | 0.61 |
| PPS | 1.2(0.94-1.53) | 0.15 | 1.16(0.91-1.48) | 0.23 | 0.85(0.67-1.09) | 0.2 | 1.07(0.84-1.36) | 0.59 | 0.91(0.64-1.3) | 0.61 | 0.69(0.48-0.99) | 0.043 |
| **Parameters** | **CLDN14** | | **CLDN15** | | **CLDN16** | | **CLDN17** | | **CLDN18** | | **CLDN19** | |
|  | **HR(95%CI)** | **p-value** | **HR(95%CI)** | **p-value** | **HR(95%CI)** | **p-value** | **HR(95%CI)** | **p-value** | **HR(95%CI)** | **p-value** | **HR(95%CI)** | **p-value** |
| RFS | 0.83(0.75-0.93) | 0.0011 | 0.78(0.7-0.87) | 9.10E-06 | 0.78(0.7-0.87) | 5.10E-06 | 0.78(0.7-0.87) | 4.90E-06 | 0.73(0.65-0.81) | 1.10E-08 | 0.81(0.69-0.94) | 0.0068 |
| OS | 1.32(1.06-1.63) | 0.012 | 0.99(0.8-1.23) | 0.94 | 0.99(0.8-1.22) | 0.9 | 1.06(0.85-1.31) | 0.61 | 0.89(0.72-1.1) | 0.29 | 1.01(0.74-1.38) | 0.95 |
| DMFS | 1.06(0.88-1.29) | 0.53 | 1.08(0.89-1.32) | 0.42 | 0.89(0.73-1.08) | 0.22 | 1.08(0.89-1.31) | 0.41 | 0.91(0.75-1.1) | 0.32 | 1.02(0.74-1.41) | 0.92 |
| PPS | 1.34(1.05-1.71) | 0.017 | 1.1(0.87-1.41) | 0.42 | 1.09(0.85-1.39) | 0.49 | 1.02(0.8-1.29) | 0.9 | 0.75(0.59-0.96) | 0.02 | 1.07(0.75-1.53) | 0.7 |
| **Parameters** | **CLDN20** | | **CLDN22** | | **CLDN23** | | **CLDN24** | |  |  |  |  |
|  | **HR(95%CI)** | **p-value** | **HR(95%CI)** | **p-value** | **HR(95%CI)** | **p-value** | **HR(95%CI)** | **p-value** |  |  |  |  |
| RFS | 0.61(0.52-0.71) | 5.90E-10 | NA | NA | 1.05(0.9-1.22) | 0.56 | NA | NA |  |  |  |  |
| OS | 1.38(1-1.9) | 0.047 | NA | NA | 1.13(0.82-1.54) | 0.45 | NA | NA |  |  |  |  |
| DMFS | 1.08(0.78-1.5) | 0.63 | NA | NA | 0.97(0.7-1.34) | 0.86 | NA | NA |  |  |  |  |
| PPS | 1.23(0.86-1.74) | 0.26 | NA | NA | 1.07(0.75-1.52) | 0.71 | NA | NA |  |  |  |  |
| **Abbreviations:** RFS, relapse-free survivaL; OS, overall survival; DMFS, distant metastasis-free survival; PPS, postprogression survival; NA, not avaliable. | | | | | | | | | | | | |
|  |  |  |  |  |  |  |  |  |  |  |  |  |
|  |  |  |  |  |  |  |  |  |  |  |  |  |
