# Supplementary material for: Comprehensive analysis of expression and prognostic value of the claudin family in human breast cancer
Source: Aging (Albany NY). 2021 Mar 10;13(6):8777–96. doi: 10.18632/aging.202687 (PMC8034964; doi:10.18632/aging.202687)
Supplement: Supplementary Table 7 [file aging-13-202687-s008.doc]

**Supplementary Table 7. Survival analyses of the claudin family with different molecular subtypes in breast cancer (Kaplan–Meier plotter).**

| **Parameters** | **CLDN1** | | **CLDN2** | | **CLDN3** | | **CLDN4** | | **CLDN5** | | **CLDN6** | |
| --- | --- | --- | --- | --- | --- | --- | --- | --- | --- | --- | --- | --- |
|  | **HR(95%CI)** | **p-value** | **HR(95%CI)** | **p-value** | **HR(95%CI)** | **p-value** | **HR(95%CI)** | **p-value** | **HR(95%CI)** | **p-value** | **HR(95%CI)** | **p-value** |
| **Basal** |  |  |  |  |  |  |  |  |  |  |  |  |
| RFS | 1.52(1.1-2.11) | 0.011 | 0.82(0.59-1.14) | 0.23 | 1.15(0.89-1.48) | 0.27 | 0.92(0.72-1.19) | 0.53 | 1.06(0.83-1.37) | 0.63 | 0.68(0.53-0.88) | 0.0031 |
| OS | 1.04(0.55-1.98) | 0.89 | 1.15(0.61-2.18) | 0.67 | 1.06(0.65-1.73) | 0.81 | 0.9(0.55-1.47) | 0.67 | 1.2(0.74-1.97) | 0.46 | 1.05(0.64-1.73) | 0.84 |
| DMFS | 1.26(0.62-2.55) | 0.53 | 0.55(0.27-1.16) | 0.11 | 0.98(0.59-1.62) | 0.92 | 0.91(0.55-1.51) | 0.72 | 0.93(0.56-1.55) | 0.79 | 0.9(0.54-1.5) | 0.68 |
| PPS | 0.85(0.37-1.93) | 0.7 | 1.47(0.64-3.42) | 0.36 | 1.36(0.76-2.44) | 0.29 | 1.02(0.57-1.82) | 0.96 | 1.13(0.63-2.04) | 0.68 | 0.92(0.51-1.66) | 0.79 |
| **Luminal A** |  |  |  |  |  |  |  |  |  |  |  |  |
| RFS | 0.71(0.56-0.91) | 0.0074 | 0.66(0.51-0.85) | 0.00095 | 1.36(1.15-1.62) | 0.00036 | 1.02(0.96-1.21) | 0.78 | 0.77(0.65-0.91) | 0.0026 | 0.67(0.56-0.8) | 4.2E-06 |
| OS | 1.04(0.55-1.98) | 0.89 | 1.15(0.61-2.18) | 0.67 | 1.06(0.65-1.73) | 0.81 | 0.9(0.55-1.47) | 0.67 | 1.2(0.74-1.97) | 0.46 | 0.79(0.55-1.12) | 0.19 |
| DMFS | 0.72(0.42-1.26) | 0.25 | 0.78(0.45-1.35) | 0.37 | 1.3(0.97-1.73) | 0.078 | 1.14(0.86-1.52) | 0.36 | 0.96(0.72-1.28) | 0.8 | 1.01(0.76-1.35) | 0.95 |
| PPS | 0.74(0.42-1.31) | 0.3 | 0.85(0.48-1.49) | 0.57 | 1.55(1.05-2.3) | 0.027 | 1.61(1.09-2.38) | 0.016 | 1.1(0.75-1.63) | 0.63 | 0.82(0.55-1.21) | 0.32 |
| **Luminal B** |  |  |  |  |  |  |  |  |  |  |  |  |
| RFS | 1.01(0.75-1.37) | 0.93 | 0.9(0.66-1.22) | 0.48 | 1.2(0.99-1.45) | 0.061 | 0.9(0.75-1.1) | 0.31 | 0.99(0.82-1.2) | 0.95 | 0.74(0.61-0.9) | 0.002 |
| OS | 0.81(0.41-1.59) | 0.54 | 0.93(0.47-1.83) | 0.83 | 1.2(0.83-1.75) | 0.33 | 1.08(0.75-1.56) | 0.68 | 0.9(0.62-1.3) | 0.57 | 1.09(0.75-1.59) | 0.65 |
| DMFS | 1.28(0.66-2.47) | 0.47 | 0.85(0.44-1.65) | 0.64 | 1.11(0.78-1.57) | 0.58 | 0.92(0.64-1.3) | 0.62 | 1.12(0.79-1.59) | 0.53 | 1.16(0.82-1.65) | 0.41 |
| PPS | 0.63(0.3-1.33) | 0.22 | 0.81(0.39-1.71) | 0.58 | 1.28(0.83-1.97) | 0.26 | 0.98(0.63-1.5) | 0.91 | 0.99(0.64-1.52) | 0.97 | 0.99(0.65-1.53) | 0.98 |
| **HER2+** |  |  |  |  |  |  |  |  |  |  |  |  |
| RFS | 0.96(0.61-1.51) | 0.87 | 0.88(0.56-1.38) | 0.57 | 1.15(0.78-1.68) | 0.48 | 1.08(0.74-1.58) | 0.7 | 1.17(0.8-1.72) | 0.42 | 0.69(0.47-1.02) | 0.06 |
| OS | 0.96(0.44-2.11) | 0.92 | 1.14(0.52-2.52) | 0.74 | 1.4(0.73-2.68) | 0.31 | 1.2(0.63-2.28) | 0.58 | 1.26(0.66-2.42) | 0.48 | 1.24(0.65-2.38) | 0.52 |
| DMFS | 1.51(0.71-3.2) | 0.28 | 1.03(0.49-2.16) | 0.94 | 1.45(0.77-2.72) | 0.24 | 1.22(0.66-2.28) | 0.52 | 1.01(0.54-1.88) | 0.97 | 1.56(0.82-2.97) | 0.17 |
| PPS | 0.77(0.33-1.8) | 0.55 | 1.04(0.45-2.42) | 0.93 | 1.14(.54-2.4) | 0.73 | 1.4(0.66-2.95) | 0.38 | 1.05(0.5-2.24) | 0.89 | 1.15(0.55-2.43) | 0.71 |
| **Parameters** | **CLDN7** | | **CLDN8** | | **CLDN9** | | **CLDN10** | | **CLDN11** | | **CLDN12** | |
|  | **HR(95%CI)** | **p-value** | **HR(95%CI)** | **p-value** | **HR(95%CI)** | **p-value** | **HR(95%CI)** | **p-value** | **HR(95%CI)** | **p-value** | **HR(95%CI)** | **p-value** |
| **Basal** |  |  |  |  |  |  |  |  |  |  |  |  |
| RFS | 1.5(1.16-1.93) | 0.0017 | 1.22(0.95-1.57) | 0.13 | 0.68(0.53-0.88) | 0.0032 | 0.77(0.6-0.99) | 0.044 | 1.25(0.91-1.74) | 0.17 | 1.2(0.87-1.66) | 0.27 |
| OS | 1.03(0.63-1.68) | 0.9 | 2.69(1.58-4.56) | 0.00014 | 0.68(0.41-1.12) | 0.13 | 0.76(0.46-1.25) | 0.28 | 1.66(0.86-3.21) | 0.13 | 1.51(0.79-2.89) | 0.21 |
| DMFS | 1.65(0.99-2.77) | 0.054 | 1.59(0.95-2.66) | 0.075 | 0.9(0.54-1.49) | 0.67 | 0.57(0.34-0.96) | 0.034 | 1.8(0.87-3.8) | 0.11 | 2.03(0.97-4.23) | 0.055 |
| PPS | 0.77(0.43-1.38) | 0.38 | 1.07(0.59-1.91) | 0.83 | 0.81(0.45-1.44) | 0.47 | 1.1(0.61-1.99) | 0.76 | 0.6(0.6-3.57) | 0.4 | 0.58(0.25-1.38) | 0.21 |
| **Luminal A** |  |  |  |  |  |  |  |  |  |  |  |  |
| RFS | 0.95(0.8-1.13) | 0.56 | 0.83(0.7-0.98) | 0.03 | 0.73(0.61-0.86) | 0.00026 | 0.7(0.59-0.83) | 4.1E-05 | 0.63(0.49-0.81) | 0.00029 | 0.82(0.64-1.05) | 0.12 |
| OS | 1.03(0.63-1.68) | 0.9 | 2.69(1.58-4.56) | 0.00014 | 0.68(0.41-1.12) | 0.13 | 0.76(0.46-1.25) | 0.28 | 1.66(0.86-3.21) | 0.13 | 1.51(0.79-2.89) | 0.21 |
| DMFS | 1.29(0.96-1.72) | 0.088 | 0.62(0.46-0.83) | 0.0012 | 1.1(0.83-1.47) | 0.51 | 1.0(0.75-1.34) | 0.98 | 0.99(0.57-1.7) | 0.96 | 0.72(0.42-1.25) | 0.24 |
| PPS | 1.33(0.9-1.96) | 0.15 | 0.99(0.67-1.46) | 0.95 | 0.86(0.58-1.27) | 0.45 | 0.86(0.59-1.28) | 0.46 | 0.95(0.54-1.66) | 0.85 | 1.17(0.66-2.09) | 0.59 |
| **Luminal B** |  |  |  |  |  |  |  |  |  |  |  |  |
| RFS | 1.14(0.94-1.38) | 1.70E-01 | 0.75(0.62-0.91) | 4.10E-03 | 0.68(0.56-0.83) | 1.00E-04 | 0.67(0.56-0.82) | 5.7E-05 | 0.71(0.52-0.96) | 0.026 | 1.03(0.76-1.4) | 0.84 |
| OS | 1.52(1.04-2.22) | 0.028 | 0.75(0.52-1.09) | 0.13 | 1.15(0.79-1.67) | 0.48 | 1.1(0.76-1.6) | 0.61 | 0.78(0.4-1.53) | 0.47 | 0.96(0.49-1.88) | 0.89 |
| DMFS | 1.22(0.86-1.74) | 0.26 | 0.76(0.53-1.09) | 0.13 | 1.17(0.82-1.67) | 0.38 | 1.15(0.81-1.63) | 0.44 | 0.74(0.38-1.44) | 0.37 | 1.36(0.7-2.63) | 0.37 |
| PPS | 1.09(0.71-1.67) | 0.71 | 0.94(0.61-1.44) | 0.76 | 1.6(1.02-2.49) | 0.037 | 1.34(0.87-2.07) | 0.19 | 0.84(0.4-1.76) | 0.65 | 0.89(0.42-1.85) | 0.75 |
| **HER2+** |  |  |  |  |  |  |  |  |  |  |  |  |
| RFS | 1.16(0.79-1.71) | 0.44 | 1.62(1.09-2.39) | 0.015 | 0.61(0.42-0.91) | 0.013 | 0.75(0.51-1.11) | 0.15 | 1.11(0.7-1.74) | 0.66 | 0.9(0.57-1.42) | 0.66 |
| OS | 1.09(0.57-2.08) | 0.8 | 1.73(0.9-3.34) | 0.097 | 0.71(0.37-1.35) | 0.29 | 1.22(0.64-2.34) | 0.55 | 0.56(0.25-1.24) | 0.15 | 0.63(0.28-1.39) | 0.25 |
| DMFS | 1.5(0.8-2.8) | 0.2 | 1.17(0.63-2.18) | 0.62 | 1.0(0.53-1.86) | 0.99 | 1.19(0.64-2.23) | 0.58 | 1.43(0.67-3.02) | 0.35 | 0.85(0.41-1.79) | 0.68 |
| PPS | 1.5(0.71-3.16) | 0.28 | 1.59(0.75-3.36) | 0.22 | 0.96(0.45-2.03) | 0.91 | 1.41(0.67-2.98) | 0.37 | 0.65(0.28-1.51) | 0.31 | 0.57(0.25-1.34) | 0.2 |
| **Parameters** | **CLDN14** | | **CLDN15** | | **CLDN16** | | **CLDN17** | | **CLDN18** | | **CLDN19** | |
|  | **HR(95%CI)** | **p-value** | **HR(95%CI)** | **p-value** | **HR(95%CI)** | **p-value** | **HR(95%CI)** | **p-value** | **HR(95%CI)** | **p-value** | **HR(95%CI)** | **p-value** |
| **Basal** |  |  |  |  |  |  |  |  |  |  |  |  |
| RFS | 0.89(0.69-1.14) | 0.36 | 0.78(0.61-1) | 0.054 | 0.68(0.52-0.87) | 0.0024 | 0.85(0.66-1.1) | 0.21 | 0.73(0.56-0.94) | 0.013 | 1.08(0.78-1.49) | 0.65 |
| OS | 1.16(0.71-1.9) | 0.56 | 0.99(0.61-1.62) | 0.97 | 1.01(0.62-1.65) | 0.97 | 1.47(0.88-2.45) | 0.13 | 0.91(0.55-1.48) | 0.69 | 0.84(0.44-1.58) | 0.58 |
| DMFS | 0.78(0.47-1.31) | 0.35 | 0.85(0.51-1.41) | 0.52 | 1.02(0.61-1.69) | 0.95 | 1.05(0.63-1.74) | 0.86 | 0.81(0.49-1.35) | 0.43 | 0.64(0.31-1.31) | 0.22 |
| PPS | 1.16(0.65-2.08) | 0.62 | 0.99(0.56-1.78) | 0.98 | 0.86(0.48-1.54) | 0.6 | 1.19(0.67-2.14) | 0.55 | 0.72(0.4-1.3) | 0.27 | 1.08(0.46-2.54) | 0.85 |
| **Luminal A** |  |  |  |  |  |  |  |  |  |  |  |  |
| RFS | 0.8(0.67-0.94) | 0.0088 | 0.75(0.63-0.89) | 0.00098 | 0.78(0.66-0.93) | 0.005 | 0.74(0.63-0.88) | 0.00069 | 0.71(0.6-0.84) | 7.3E-05 | 0.7(0.55-0.9) | 0.0047 |
| OS | 1.16(0.71-1.9) | 0.56 | 0.99(0.61-1.62) | 0.97 | 1.01(0.62-1.65) | 0.97 | 1.47(0.88-2.45) | 0.13 | 0.91(0.55-1.48) | 0.69 | 0.84(0.44-1.58) | 0.58 |
| DMFS | 1.15(0.87-1.54) | 0.33 | 1.19(0.89-1.6) | 0.23 | 0.94(0.71-1.26) | 0.69 | 1.05(0.79-1.39) | 0.76 | 0.89(0.67-1.19) | 0.45 | 1.45(0.83-2.51) | 0.19 |
| PPS | 1.27(0.86-1.87) | 0.22 | 1.13(0.77-1.68) | 0.53 | 1.24(0.84-1.83) | 0.27 | 0.81(0.55-1.2) | 0.29 | 0.65(0.44-0.96) | 0.03 | 0.98(0.56-1.72) | 0.96 |
| **Luminal B** |  |  |  |  |  |  |  |  |  |  |  |  |
| RFS | 0.87(0.72-1.05) | 0.14 | 0.82(0.67-0.99) | 0.038 | 0.71(0.59-0.86) | 0.00049 | 0.73(0.6-0.89) | 0.0015 | 0.78(0.65-0.95) | 0.012 | 0.92(0.67-1.24) | 0.57 |
| OS | 1.5(1.03-2.19) | 0.033 | 0.97(0.67-1.41) | 0.86 | 0.91(0.63-1.33) | 0.63 | 0.99(0.68-1.44) | 0.97 | 0.98(0.68-1.43) | 0.93 | 1.82(0.91-3.66) | 0.086 |
| DMFS | 1.1(0.78-1.57) | 0.59 | 1.04(0.73-1.47) | 0.84 | 0.76(0.53-1.08) | 0.12 | 1.33(0.94-1.9) | 0.11 | 1.31(0.92-1.87) | 0.13 | 1.69(0.86-3.31) | 0.12 |
| PPS | 1.38(0.9-2.13) | 0.14 | 1.09(0.71-1.68) | 0.69 | 1.04(0.68-1.6) | 0.85 | 0.97(0.63-1.5) | 0.89 | 1.09(0.71-1.67) | 0.7 | 0.66(0.32-1.4) | 0.28 |
| **HER2+** |  |  |  |  |  |  |  |  |  |  |  |  |
| RFS | 0.67(0.45-0.98) | 0.039 | 1.03(0.7-1.5) | 0.9 | 1.2(0.82-1.77) | 0.34 | 0.7(0.48-1.03) | 0.072 | 0.68(0.46-1.01) | 0.052 | 1.27(0.81-2.0) | 0.3 |
| OS | 1.12(0.59-2.14) | 0.73 | 1.88(0.97-3.66) | 0.058 | 1.36(0.71-2.6) | 0.36 | 1.04(0.55-1.99) | 0.9 | 1.03(0.54-1.96) | 0.93 | 1.45(0.65-3.23) | 0.36 |
| DMFS | 1.26(0.67-2.35) | 0.47 | 1.43(0.76-2.68) | 0.26 | 1.1(0.59-2.06) | 0.76 | 1.06(0.57-1.98) | 0.85 | 0.81(0.44-1.52) | 0.51 | 1.73(0.81-3.71) | 0.15 |
| PPS | 1.4(0.66-2.96) | 0.38 | 2.19(1.02-4.72) | 0.041 | 1.57(0.74-3.33) | 0.24 | 1.13(0.54-2.38) | 0.75 | 0.99(0.47-2.08) | 0.98 | 1.64(0.7-3.83) | 0.25 |
| **Parameters** | **CLDN20** | | **CLDN22** | | **CLDN23** | | **CLDN24** | |  |  |  |  |
|  | **HR(95%CI)** | **p-value** | **HR(95%CI)** | **p-value** | **HR(95%CI)** | **p-value** | **HR(95%CI)** | **p-value** |  |  |  |  |
| **Basal** |  |  |  |  |  |  |  |  |  |  |  |  |
| RFS | 0.55(0.39-0.77) | 0.0004 | NA | NA | 0.91(0.66-1.26) | 0.59 | NA | NA |  |  |  |  |
| OS | 1.35(0.71-2.57) | 0.36 | NA | NA | 0.51(0.51-1.83) | 0.92 | NA | NA |  |  |  |  |
| DMFS | 1.08(0.53-2.18) | 0.84 | NA | NA | 0.71(0.35-1.45) | 0.35 | NA | NA |  |  |  |  |
| PPS | 3.96(1.53-10.25) | 0.0024 | NA | NA | 0.95(0.39-2.31) | 0.91 | NA | NA |  |  |  |  |
| **Luminal A** |  |  |  |  |  |  |  |  |  |  |  |  |
| RFS | 0.63(0.49-0.81) | 0.00025 | NA | NA | 1.07(0.83-1.36) | 0.61 | NA | NA |  |  |  |  |
| OS | 1.35(0.71-2.57) | 0.36 | NA | NA | 0.97(0.51-1.83) | 0.92 | NA | NA |  |  |  |  |
| DMFS | 1.05(0.61-1.81) | 0.86 | NA | NA | 1.28(0.74-2.22) | 0.37 | NA | NA |  |  |  |  |
| PPS | 1.06(0.6-1.87) | 0.84 | NA | NA | 0.76(0.43-1.33) | 0.34 | NA | NA |  |  |  |  |
| **Luminal B** |  |  |  |  |  |  |  |  |  |  |  |  |
| RFS | 0.61(0.44-0.83) | 0.0018 | NA | NA | 0.65(0.47-0.88) | 0.0054 | NA | NA |  |  |  |  |
| OS | 0.99(0.5-1.94) | 0.97 | NA | NA | 1.09(0.55-2.15) | 0.8 | NA | NA |  |  |  |  |
| DMFS | 1.25(0.65-2.41) | 0.5 | NA | NA | 0.51(0.25-1.03) | 0.055 | NA | NA |  |  |  |  |
| PPS | 0.83(0.4-1.73) | 0.62 | NA | NA | 1.1(0.53-2.29) | 0.8 | NA | NA |  |  |  |  |
| **HER2+** |  |  |  |  |  |  |  |  |  |  |  |  |
| RFS | 0.9(0.57-1.43) | 0.66 | NA | NA | 0.72(0.46-1.14) | 0.17 | NA | NA |  |  |  |  |
| OS | 2.45(1.06-5.69) | 0.031 | NA | NA | 0.99(0.45-2.18) | 0.99 | NA | NA |  |  |  |  |
| DMFS | 1.25(0.59-2.67) | 0.55 | NA | NA | 0.56(0.26-1.21) | 0.14 | NA | NA |  |  |  |  |
| PPS | 2.44(1.02-5.81) | 0.039 | NA | NA | 1.81(0.78-4.21) | 0.16 | NA | NA |  |  |  |  |
| **Abbreviations:** RFS, relapse-free survivaL; OS, overall survival; DMFS, distant metastasis-free survival; PPS, postprogression survival; NA, not avaliable. | | | | | | | | | | | | |
|  |  |  |  |  |  |  |  |  |  |  |  |  |
|  |  |  |  |  |  |  |  |  |  |  |  |  |
