# Supplementary material for: Comprehensive analysis of expression and prognostic value of the claudin family in human breast cancer
Source: Aging (Albany NY). 2021 Mar 10;13(6):8777–96. doi: 10.18632/aging.202687 (PMC8034964; doi:10.18632/aging.202687)
Supplement: Supplementary Table 8 [file aging-13-202687-s009.doc]

**Supplementary Table 8. Survival analyses of the claudin family with different lymph node status in breast cancer (Kaplan–Meier plotter).**

| **Parameters** | **CLDN1** | | **CLDN2** | | **CLDN3** | | **CLDN4** | | **CLDN5** | | **CLDN6** | |
| --- | --- | --- | --- | --- | --- | --- | --- | --- | --- | --- | --- | --- |
|  | **HR(95%CI)** | **p-value** | **HR(95%CI)** | **p-value** | **HR(95%CI)** | **p-value** | **HR(95%CI)** | **p-value** | **HR(95%CI)** | **p-value** | **HR(95%CI)** | **p-value** |
| **Lymph node postive** |  |  |  |  |  |  |  |  |  |  |  |  |
| RFS | 1.16(0.9-1.49) | 0.24 | 0.95(0.74-1.22) | 0.69 | 1.05(0.86-1.28) | 0.61 | 1.03(0.84-1.25) | 0.8 | 0.84(0.69-1.03) | 0.087 | 1.16(0.95-1.41) | 0.14 |
| OS | 0.85(0.5-1.45) | 0.56 | 0.97(0.57-1.64) | 0.91 | 1.14(0.77-1.68) | 0.5 | 0.91(0.62-1.34) | 0.63 | 1.23(0.83-1.81) | 0.3 | 1.25(0.84-1.84) | 0.27 |
| DMFS | 1.68(0.9-3.14) | 0.1 | 0.67(0.36-1.25) | 0.21 | 1.2(0.81-1.77) | 0.36 | 0.92(0.62-1.35) | 0.66 | 0.96(0.65-1.42) | 0.85 | 1.13(0.77-1.67) | 0.53 |
| PPS | 0.53(0.28-1.01) | 0.049 | 0.78(0.42-1.44) | 0.42 | 1.42(0.91-2.22) | 0.12 | 1.18(0.75-1.84) | 0.47 | 1.18(0.76-1.85) | 0.46 | 2.12(1.35-3.33) | 0.0009 |
| **Lymph node negative** |  |  |  |  |  |  |  |  |  |  |  |  |
| RFS | 1.07(0.73-1.57) | 0.74 | 0.99(0.67-1.45) | 0.95 | 1.1(0.93-1.3) | 0.26 | 0.98(0.83-1.16) | 0.78 | 0.87(0.74-1.03) | 0.11 | 0.98(0.83-1.16) | 0.81 |
| OS | 2.25(0.88-5.73) | 0.082 | 1.1(0.45-2.72) | 0.83 | 1.6(1.09-2.35) | 0.015 | 1.32(0.91-1.92) | 0.14 | 0.79(0.54-1.14) | 0.21 | 0.79(0.54-1.15) | 0.22 |
| DMFS | 1.88(0.77-4.55) | 0.16 | 0.53(0.22-1.29) | 0.15 | 1.51(1.14-1.99) | 0.0033 | 1.23(0.94-1.61) | 0.13 | 0.82(0.62-1.08) | 0.15 | 0.95(0.73-1.25) | 0.74 |
| PPS | 1.3(0.42-4.02) | 0.64 | 1.24(0.41-3.73) | 0.7 | 1.95(1.25-3.06) | 0.003 | 1.02(0.67-1.56) | 0.93 | 0.94(0.61-1.44) | 0.77 | 0.6(0.39-0.92) | 0.018 |
| **Parameters** | **CLDN7** | | **CLDN8** | | **CLDN9** | | **CLDN10** | | **CLDN11** | | **CLDN12** | |
|  | **HR(95%CI)** | **p-value** | **HR(95%CI)** | **p-value** | **HR(95%CI)** | **p-value** | **HR(95%CI)** | **p-value** | **HR(95%CI)** | **p-value** | **HR(95%CI)** | **p-value** |
| **Lymph node postive** |  |  |  |  |  |  |  |  |  |  |  |  |
| RFS | 0.98(0.81-1.2) | 0.88 | 1.03(0.85-1.26) | 0.74 | 1.06(0.87-1.29) | 0.56 | 1.04(0.85-1.26) | 0.73 | 0.74(0.58-0.95) | 0.019 | 0.81(0.63-1.04) | 0.096 |
| OS | 0.98(0.67-1.45) | 0.94 | 1.28(0.87-1.89) | 0.21 | 0.29(0.87-1.9) | 0.2 | 1.02(0.69-1.49) | 0.94 | 0.99(0.59-1.68) | 0.98 | 0.62(0.36-1.05) | 0.074 |
| DMFS | 1.03(0.7-1.51) | 0.9 | 1.57(1.06-2.31) | 0.023 | 1.17(0.79-1.72) | 0.44 | 1.07(0.73-1.57) | 0.73 | 0.8(0.43-1.48) | 0.48 | 1.05(0.56-1.95) | 0.88 |
| PPS | 1.25(0.8-1.95) | 0.33 | 1.22(0.78-1.92) | 0.38 | 1.94(1.24-3.06) | 0.0035 | 1.67(1.07-2.62) | 0.023 | 0.94(0.51-1.75) | 0.86 | 0.57(0.31-1.06) | 0.072 |
| **Lymph node negative** |  |  |  |  |  |  |  |  |  |  |  |  |
| RFS | 0.86(0.73-1.02) | 0.087 | 0.85(0.72-1) | 0.052 | 0.89(0.75-1.05) | 0.17 | 1.06(0.9-1.26) | 0.48 | 0.78(0.53-1.15) | 0.21 | 0.94(0.64-1.39) | 0.76 |
| OS | 1.43(0.98-2.07) | 0.06 | 0.8(0.55-1.17) | 0.25 | 0.58(0.4-0.86) | 0.0059 | 0.91(0.62-1.33) | 0.62 | 1.11(0.45-2.75) | 0.81 | 0.54(0.22-1.35) | 0.18 |
| DMFS | 1.17(0.89-1.53) | 0.27 | 0.62(0.47-0.82) | 0.0007 | 0.89(0.68-1.17) | 0.42 | 1.09(0.83-1.43) | 0.54 | 1.58(0.65-3.81) | 0.31 | 0.81(0.34-1.92) | 0.63 |
| PPS | 1.19(0.77-1.82) | 0.44 | 1.09(0.71-1.67) | 0.71 | 0.46(0.3-0.72) | 0.0005 | 0.91(0.59-1.39) | 0.65 | 0.66(0.22-1.99) | 0.46 | 0.4(0.12-1.31) | 0.12 |
| **Parameters** | **CLDN14** | | **CLDN15** | | **CLDN16** | | **CLDN17** | | **CLDN18** | | **CLDN19** | |
|  | **HR(95%CI)** | **p-value** | **HR(95%CI)** | **p-value** | **HR(95%CI)** | **p-value** | **HR(95%CI)** | **p-value** | **HR(95%CI)** | **p-value** | **HR(95%CI)** | **p-value** |
| **Lymph node postive** |  |  |  |  |  |  |  |  |  |  |  |  |
| RFS | 0.95(0.78-1.16) | 0.6 | 0.91(0.75-1.11) | 0.34 | 1.0(0.82-1.22) | 0.99 | 1.05(0.86-1.28) | 0.62 | 0.94(0.77-1.14) | 0.53 | 1.09(0.85-1.4) | 0.5 |
| OS | 1.32(0.89-1.94) | 0.16 | 1.08(0.73-1.59) | 0.7 | 1.25(0.85-1.84) | 0.26 | 1.04(.71-1.53) | 0.84 | 1.33(0.9-1.97) | 0.15 | 1.1(0.65-1.86) | 0.72 |
| DMFS | 0.93(0.63-1.36) | 0.7 | 1.27(0.86-1.87) | 0.23 | 1.01(0.69-1.48) | 0.96 | 1.43(0.97-2.12) | 0.068 | 0.97(0.66-1.42) | 0.87 | 1.26(0.68-2.34) | 0.46 |
| PPS | 1.57(1-2.48) | 0.049 | 1.39(0.89-2.18) | 0.14 | 1.07(0.69-1.67) | 0.77 | 1.67(1.07-2.62) | 0.024 | 1.84(1.17-2.89) | 0.007 | 1.21(0.65-2.24) | 0.54 |
| **Lymph node negative** |  |  |  |  |  |  |  |  |  |  |  |  |
| RFS | 1.0(0.84-1.18) | 0.96 | 0.93(0.78-1.1) | 0.38 | 0.89(0.75-1.05) | 0.18 | 0.9(0.76-1.06) | 0.21 | 1.01(0.85-1.2) | 0.9 | 0.83(0.57-1.23) | 0.35 |
| OS | 1.08(0.75-1.57) | 0.67 | 0.85(0.58-1.24) | 0.4 | 0.79(0.54-1.15) | 0.22 | 0.89(0.62-1.3) | 0.56 | 0.8(0.55-1.16) | 0.24 | 0.58(0.23-1.45) | 0.24 |
| DMFS | 1.03(0.79-1.35) | 0.84 | 0.93(0.71-1.23) | 0.63 | 0.92(0.7-1.2) | 0.53 | 1.03(0.79-1.35) | 0.82 | 1.18(0.9-1.55) | 0.23 | 0.74(0.31-1.75) | 0.49 |
| PPS | 1.31(0.85-2.01) | 0.21 | 0.84(0.54-1.29) | 0.42 | 0.97(0.63-1.49) | 0.89 | 0.86(0.56-1.31) | 0.47 | 0.57(0.37-0.87) | 0.0088 | 0.26(0.07-0.98) | 0.034 |
| **Parameters** | **CLDN20** | | **CLDN22** | | **CLDN23** | | **CLDN24** | |  |  |  |  |
|  | **HR(95%CI)** | **p-value** | **HR(95%CI)** | **p-value** | **HR(95%CI)** | **p-value** | **HR(95%CI)** | **p-value** |  |  |  |  |
| **Lymph node postive** |  |  |  |  |  |  |  |  |  |  |  |  |
| RFS | 0.71(0.55-0.92) | 0.0088 | NA | NA | 0.93(0.72-1.19) | 0.55 | NA | NA |  |  |  |  |
| OS | 1.74(1.02-2.96) | 0.04 | NA | NA | 1.08(0.64-1.83) | 0.77 | NA | NA |  |  |  |  |
| DMFS | 1.05(0.57-1.94) | 0.88 | NA | NA | 0.76(0.41-1.41) | 0.38 | NA | NA |  |  |  |  |
| PPS | 2.26(1.21-4.23) | 0.0091 | NA | NA | 1.37(0.74-2.54) | 0.31 | NA | NA |  |  |  |  |
| **Lymph node negative** |  |  |  |  |  |  |  |  |  |  |  |  |
| RFS | 0.81(0.55-1.2) | 0.29 | NA | NA | 1.2(0.82-1.76) | 0.35 | NA | NA |  |  |  |  |
| OS | 1.19(0.48-2.98) | 0.7 | NA | NA | 0.94(0.38-2.35) | 0.9 | NA | NA |  |  |  |  |
| DMFS | 0.47(0.19-1.16) | 0.093 | NA | NA | 2.31(0.93-5.73) | 0.064 | NA | NA |  |  |  |  |
| PPS | 1.0(0.33-3.01) | 1 | NA | NA | 0.36(0.09-1.42) | 0.13 | NA | NA |  |  |  |  |
| **Abbreviations:** RFS, relapse-free survivaL; OS, overall survival; DMFS, distant metastasis-free survival; PPS, postprogression survival; NA, not avaliable. | | | | | | | | | | | | |
|  |  |  |  |  |  |  |  |  |  |  |  |  |
