# Supplementary material for: Comprehensive analysis of expression and prognostic value of the claudin family in human breast cancer
Source: Aging (Albany NY). 2021 Mar 10;13(6):8777–96. doi: 10.18632/aging.202687 (PMC8034964; doi:10.18632/aging.202687)
Supplement: Supplementary Table 9 [file aging-13-202687-s010.doc]

**Supplementary Table 9. Survival analyses of the claudin family with different histologic grades in breast cancer (Kaplan–Meier plotter).**

| **Parameters** | **CLDN1** | | **CLDN2** | | **CLDN3** | | **CLDN4** | | **CLDN5** | | **CLDN6** | |
| --- | --- | --- | --- | --- | --- | --- | --- | --- | --- | --- | --- | --- |
|  | **HR(95%CI)** | **p-value** | **HR(95%CI)** | **p-value** | **HR(95%CI)** | **p-value** | **HR(95%CI)** | **p-value** | **HR(95%CI)** | **p-value** | **HR(95%CI)** | **p-value** |
| **Grade 1** |  |  |  |  |  |  |  |  |  |  |  |  |
| RFS | 0.36(0.11-1.14) | 0.069 | 0.75(0.26-2.17) | 0.6 | 1.02(0.6-1.7) | 0.95 | 0.74(0.44-1.25) | 0.26 | 0.65(0.39-1.1) | 0.11 | 1.08(0.664-1.81) | 0.78 |
| OS | 0(0-inf) | 0.081 | 2.17(0.19-24.62) | 0.52 | 0.72(0.29-1.79) | 0.48 | 0.68(0.27-1.69) | 0.4 | 0.79(0.33-1.9) | 0.59 | 0.56(0.22-1.41) | 0.21 |
| DMFS | NA |  | NA |  | 2.07(0.85-5.06) | 0.1 | 1.24(0.53-2.89) | 0.62 | 0.87(0.38-1.97) | 0.73 | 1.43(0.63-3.27) | 0.39 |
| PPS | 0.43(0.04-4.84) | 0.49 | 0.57(0.05-6.33) | 0.64 | 0.76(0.26-2.19) | 0.61 | 0.45(0.15-1.34) | 0.14 | 0.81(0.29-2.22) | 0.68 | 0.53(0.19-1.47) | 0.21 |
| **Grade 2** |  |  |  |  |  |  |  |  |  |  |  |  |
| RFS | 1.25(0.75-2.08) | 0.39 | 0.88(0.53-1.46) | 0.62 | 1.24(0.97-1.58) | 0.081 | 0.9(0.71-1.14) | 0.38 | 0.95(0.74-1.2) | 0.65 | 0.86(0.67-1.09) | 0.2 |
| OS | 0.76(0.24-2.41) | 0.65 | 1.62(0.51-5.13) | 0.41 | 1.89(1.21-2.97) | 0.0046 | 1.76(1.14-2.74) | 0.01 | 0.93(0.61-1.43) | 0.75 | 0.83(0.54-1.29) | 0.41 |
| DMFS | 0.68(0.24-1.92) | 0.47 | 0.43(0.15-1.26) | 0.11 | 1.5(1.06-2.14) | 0.022 | 1.22(0.87-1.72) | 0.25 | 1.14(0.81-1.62) | 0.44 | 0.93(0.66-1.31) | 0.66 |
| PPS | 0.95(0.17-5.34) | 0.95 | 0.75(0.15-3.86) | 0.73 | 1.99(1.2-3.31) | 0.0065 | 1.94(1.18-3.18) | 0.0075 | 1.18(0.73-1.92) | 0.5 | 1.13(0.7-1.83) | 0.62 |
| **Grade 3** |  |  |  |  |  |  |  |  |  |  |  |  |
| RFS | 1.41(1.03-1.92) | 0.032 | 0.87(0.64-1.18) | 0.37 | 1.29(1.04-1.6) | 0.023 | 1.01(0.81-1.25) | 0.96 | 1.14(0.92-1.42) | 0.24 | 0.87(0.7-1.09) | 0.22 |
| OS | 1.41(0.84-2.34) | 0.19 | 0.95(0.57-1.58) | 0.84 | 1.39(1-1.93) | 0.051 | 1.22(0.88-1.69) | 0.23 | 1.19(0.86-1.65) | 0.29 | 0.9(0.65-1.25) | 0.52 |
| DMFS | 1.96(1.08-3.55) | 0.025 | 0.65(0.36-1.16) | 0.14 | 1.26(0.89-1.79) | 0.19 | 1.15(0.82-1.63) | 0.42 | 1.06(0.75-1.5) | 0.73 | 0.99(0.7-1.4) | 0.94 |
| PPS | 1.07(0.59-1.94) | 0.82 | 0.89(0.5-1.6) | 0.7 | 1.02(0.69-1.49) | 0.93 | 1.01(0.69-1.47) | 0.97 | 1.08(0.73-1.59) | 0.7 | 0.86(0.58-1.28) | 0.46 |
| **Parameters** | **CLDN7** | | **CLDN8** | | **CLDN9** | | **CLDN10** | | **CLDN11** | | **CLDN12** | |
|  | **HR(95%CI)** | **p-value** | **HR(95%CI)** | **p-value** | **HR(95%CI)** | **p-value** | **HR(95%CI)** | **p-value** | **HR(95%CI)** | **p-value** | **HR(95%CI)** | **p-value** |
| **Grade 1** |  |  |  |  |  |  |  |  |  |  |  |  |
| RFS | 0.88(0.52-1.48) | 0.62 | 0.96(0.57-1.61) | 0.88 | 0.96(0.57-1.6) | 0.86 | 1.15(0.68-1.92) | 0.6 | 0.25(0.07-0.91) | 0.023 | 1.2(0.42-3.47) | 0.73 |
| OS | 1.16(0.45-2.95) | 0.76 | 0.86(0.35-2.11) | 0.74 | 0.92(0.37-2.25) | 0.85 | 1.27(0.49-3.29) | 0.63 | 0(0-inf) | 0.081 | 0.41(0.04-4.56) | 0.45 |
| DMFS | 1.27(0.55-2.96) | 0.58 | 1.77(0.75-4.18) | 0.19 | 1.57(0.69-3.6) | 0.28 | 1.41(0.62-3.21) | 0.41 | NA |  | NA |  |
| PPS | 1.33(0.46-3.84) | 0.6 | 0.64(0.23-1.77) | 0.39 | 0.57(0.19-1.65) | 0.29 | 0.77(0.28-2.13) | 0.62 | 0.43(0.04-4.84) | 0.49 | 0.57(0.05-6.33) | 0.64 |
| **Grade 2** |  |  |  |  |  |  |  |  |  |  |  |  |
| RFS | 0.87(0.68-1.1) | 0.24 | 0.82(0.65-1.05) | 0.11 | 0.82(0.64-1.05) | 0.11 | 0.84(0.66-1.07) | 0.15 | 0.71(0.43-1.19) | 0.2 | 0.72(0.43-1.19) | 0.2 |
| OS | 1.2(0.79-1.85) | 0.39 | 0.74(0.48-1.14) | 0.18 | 0.77(0.5-1.2) | 0.25 | 1.2(0.78-1.85) | 0.41 | 0.71(0.23-2.25) | 0.56 | 0.23(0.06-0.87) | 0.019 |
| DMFS | 1.15(0.81-1.62) | 0.43 | 0.85(0.6-1.2) | 0.36 | 1.0(0.71-1.42) | 1 | 1.02(0.72-1.5-44) | 0.93 | 0.91(0.33-2.53) | 0.86 | 0.51(0.18-1.45) | 0.2 |
| PPS | 1.41(0.87-2.3) | 0.16 | 0.89(0.55-1.45) | 0.64 | 1.35(0.83-2.19) | 0.23 | 1.94(1.19-3.17) | 0.0072 | 0.27(0.05-1.48) | 0.11 | 0.13(0.01-1.15) | 0.032 |
| **Grade 3** |  |  |  |  |  |  |  |  |  |  |  |  |
| RFS | 1.29(1.04-1.6) | 0.022 | 1.02(0.82-1.27) | 0.86 | 0.85(0.68-1.06) | 0.15 | 0.88(0.71-1.09) | 0.24 | 1.19(0.87-1.62) | 0.27 | 1.23(0.9-1.68) | 0.2 |
| OS | 1.29(0.93-1.8) | 0.12 | 1.24(0.89-1.72) | 0.2 | 1.0(0.72-1.38) | 0.99 | 1.1(0.79-1.52) | 0.58 | 1.61(0.96-2.72) | 0.069 | 0.65(0.39-1.09) | 0.1 |
| DMFS | 1.52(1.07-2.16) | 0.018 | 0.81(0.57-1.15) | 0.25 | 1.01(0.72-1.43) | 0.94 | 1.02(0.72-1.44) | 0.9 | 1.31(0.74-2.34) | 0.36 | 1.31(0.73-2.34) | 0.36 |
| PPS | 0.82(0.56-1.2) | 0.3 | 1.55(1.06-2.27) | 0.023 | 1.03(0.71-1.51) | 0.87 | 1.12(0.77-1.65) | 0.55 | 1.39(0.77-2.51) | 0.27 | 0.57(0.32-1.04) | 0.063 |
| **Parameters** | **CLDN14** | | **CLDN15** | | **CLDN16** | | **CLDN17** | | **CLDN18** | | **CLDN19** | |
|  | **HR(95%CI)** | **p-value** | **HR(95%CI)** | **p-value** | **HR(95%CI)** | **p-value** | **HR(95%CI)** | **p-value** | **HR(95%CI)** | **p-value** | **HR(95%CI)** | **p-value** |
| **Grade 1** |  |  |  |  |  |  |  |  |  |  |  |  |
| RFS | 1.02(0.6-1.71) | 0.95 | 0.85(0.5-1.43) | 0.54 | 1.02(0.61-1.72) | 0.94 | 1.13(0.68-1.9) | 0.64 | 0.56(0.56-1.58) | 0.82 | 0.6(0.2-1.78) | 0.35 |
| OS | 1.22(0.5-2.99) | 0.66 | 0.97(0.4-2.35) | 0.95 | 0.89(0.34-2.33) | 0.81 | 0.41(0.15-1.14) | 0.077 | 0.85(0.35-2.09) | 0.72 | 0.57(0.05-6.27) | 0.64 |
| DMFS | 0.77(0.32-1.83) | 0.55 | 1.87(0.8-4.37) | 0.14 | 1.06(0.46-2.48) | 0.89 | 1.18(0.51-2.71) | 0.7 | 1.2(0.52-2.78) | 0.66 | NA |  |
| PPS | 1.71(0.63-4.61) | 0.29 | 1.12(0.41-3.05) | 0.83 | 0.43(0.15-1.24) | 0.11 | 0.38(0.13-1.11) | 0.066 | 1.11(0.41-3.0) | 0.84 | 0.57(0.05-6.33) | 0.64 |
| **Grade 2** |  |  |  |  |  |  |  |  |  |  |  |  |
| RFS | 0.91(0.71-1.15) | 0.42 | 1.02(0.8-1.29) | 0.89 | 0.87(0.68-1.1) | 0.24 | 1.12(0.88-1.43) | 0.35 | 0.91(0.72-1.16) | 0.45 | 0.83(0.5-1.37) | 0.46 |
| OS | 1.3(0.84-1.99) | 0.23 | 0.9(0.59-1.39) | 0.65 | 1.21(0.79-1.86) | 0.37 | 1.45(0.94-2.24) | 0.087 | 1.04(0.68-1.61) | 0.85 | 1.15(0.36-3.65) | 0.81 |
| DMFS | 0.95(0.67-1.34) | 0.75 | 1.32(0.93-1.87) | 0.12 | 0.89(0.63-1.25) | 0.5 | 1.23(0.87-1.74) | 0.24 | 1.18(0.84-1.67) | 0.34 | 1.79(0.64-5.06) | 0.26 |
| PPS | 1.46(0.89-2.4) | 0.13 | 0.81(0.5-1.31) | 0.38 | 1.65(1.01-2.69) | 0.043 | 1.36(0.84-2.21) | 0.21 | 1.84(1.13-3) | 0.013 | 1.06(0.19-5.98) | 0.95 |
| **Grade 3** |  |  |  |  |  |  |  |  |  |  |  |  |
| RFS | 1.14(0.91-1.41) | 0.25 | 1.17(0.94-1.45) | 0.16 | 0.8(0.64-1.0) | 0.046 | 0.99(0.8-1.23) | 0.93 | 0.92(0.74-1.15) | 0.48 | 1.25(0.91-1.7) | 0.16 |
| OS | 1.33(0.96-1.85) | 0.09 | 1.38(0.99-1.91) | 0.058 | 1.05(0.76-1.46) | 0.76 | 0.97(0.7-1.35) | 0.87 | 0.95(0.69-1.32) | 0.77 | 1.35(0.81-2.25) | 0.25 |
| DMFS | 0.94(0.67-1.33) | 0.74 | 1.03(0.73-1.46) | 0.86 | 0.93(0.66-1.32) | 0.69 | 1.13(0.8-1.6) | 0.49 | 0.87(0.61-1.22) | 0.41 | 0.82(0.46-1.45) | 0.449 |
| PPS | 1.44(0.98-2.12) | 0.064 | 1.1(0.75-1.62) | 0.61 | 1.01(0.69-1.48) | 0.97 | 1.26(0.86-1.85) | 0.23 | 0.77(0.52-1.12) | 0.17 | 1.05(0.58-1.89) | 0.87 |
| **Parameters** | **CLDN20** | | **CLDN22** | | **CLDN23** | | **CLDN24** | |  |  |  |  |
|  | **HR(95%CI)** | **p-value** | **HR(95%CI)** | **p-value** | **HR(95%CI)** | **p-value** | **HR(95%CI)** | **p-value** |  |  |  |  |
| **Grade 1** |  |  |  |  |  |  |  |  |  |  |  |  |
| RFS | 0.54(0.18-1.61) | 0.26 | NA | NA | 2.83(0.89-9.03) | 0.067 | NA | NA |  |  |  |  |
| OS | 1.76(0.16-19.53) | 0.64 | NA | NA | 2.87(0.26-31.76) | 0.37 | NA | NA |  |  |  |  |
| DMFS | NA |  | NA | NA | NA |  | NA | NA |  |  |  |  |
| PPS | 3.41(0.29-39.81) | 0.3 | NA | NA | 3.41(0.29-39.81) | 0.3 | NA | NA |  |  |  |  |
| **Grade 2** |  |  |  |  |  |  |  |  |  |  |  |  |
| RFS | 0.72(0.43-1.21) | 0.21 | NA | NA | 1.06(0.64-1.76) | 0.81 | NA | NA |  |  |  |  |
| OS | 3.22(0.87-11.92) | 0.063 | NA | NA | 2.21(0.67-7.35) | 0.18 | NA | NA |  |  |  |  |
| DMFS | 1.89(0.67-5.36) | 0.22 | NA | NA | 0.52(0.18-1.54) | 0.23 | NA | NA |  |  |  |  |
| PPS | 5.75(0.67-49.47) | 0.072 | NA | NA | 0.58(0.08-4.21) | 0.59 | NA | NA |  |  |  |  |
| **Grade 3** |  |  |  |  |  |  |  |  |  |  |  |  |
| RFS | 0.97(0.71-1.33) | 0.87 | NA | NA | 0.94(0.69-1.28) | 0.7 | NA | NA |  |  |  |  |
| OS | 1.49(0.89-2.48) | 0.12 | NA | NA | 1.15(0.69-1.9) | 0.6 | NA | NA |  |  |  |  |
| DMFS | 0.98(0.55-1.74) | 0.95 | NA | NA | 0.97(0.55-1.74) | 0.93 | NA | NA |  |  |  |  |
| PPS | 1.76(0.97-3.19) | 0.058 | NA | NA | 1.48(0.82-2.66) | 0.19 | NA | NA |  |  |  |  |
| **Abbreviations:** RFS, relapse-free survivaL; OS, overall survival; DMFS, distant metastasis-free survival; PPS, postprogression survival; NA, not avaliable. | | | | | | | | | | | | |
|  |  |  |  |  |  |  |  |  |  |  |  |  |
